# An ethnically relevant consensus Korean reference genome is a step towards personal reference genomes

**DOI:** 10.1038/ncomms13637

**Published:** 2016-11-24

**Authors:** Yun Sung Cho, Hyunho Kim, Hak-Min Kim, Sungwoong Jho, JeHoon Jun, Yong Joo Lee, Kyun Shik Chae, Chang Geun Kim, Sangsoo Kim, Anders Eriksson, Jeremy S. Edwards, Semin Lee, Byung Chul Kim, Andrea Manica, Tae-Kwang Oh, George M. Church, Jong Bhak

**Affiliations:** 1 The Genomics Institute (TGI), Ulsan National Institute of Science and Technology (UNIST), Ulsan 44919, Korea; 2 Department of Biomedical Engineering, School of Life Sciences, Ulsan National Institute of Science and Technology (UNIST), Ulsan 44919, Korea; 3 Personal Genomics Institute, Genome Research Foundation, Cheongju 28160, Korea; 4 Geromics Inc., Ulsan National Institute of Science and Technology (UNIST), Ulsan 44919, Korea; 5 National Standard Reference Center, Korea Research Institute of Standards and Science, Daejeon 34113, Korea; 6 School of Systems Biomedical Science, Soongsil University, Seoul 06978, Korea; 7 Department of Zoology, University of Cambridge, Downing Street, Cambridge CB2 3EJ, UK; 8 Chemistry and Chemical Biology, UNM Comprehensive Cancer Center, University of New Mexico, Albuquerque, New Mexico 87131, USA; 9 Infection and Immunity Research Center, Korea Research Institute of Bioscience and Biotechnology, Daejeon 34141, Korea; 10 Department of Genetics, New Research Building (NRB), Harvard Medical School, 77 Avenue Louis Pasteur, Room 238, Boston, Massachusetts 02115, USA

## Abstract

Human genomes are routinely compared against a universal reference. However, this strategy could miss population-specific and personal genomic variations, which may be detected more efficiently using an ethnically relevant or personal reference. Here we report a hybrid assembly of a Korean reference genome (KOREF) for constructing personal and ethnic references by combining sequencing and mapping methods. We also build its consensus variome reference, providing information on millions of variants from 40 additional ethnically homogeneous genomes from the Korean Personal Genome Project. We find that the ethnically relevant consensus reference can be beneficial for efficient variant detection. Systematic comparison of human assemblies shows the importance of assembly quality, suggesting the necessity of new technologies to comprehensively map ethnic and personal genomic structure variations. In the era of large-scale population genome projects, the leveraging of ethnicity-specific genome assemblies as well as the human reference genome will accelerate mapping all human genome diversity.

## Abstract (web summary)

The utility of a universal reference sequence for human genome comparisons is dependent on the ethnic origins of the individuals being sequenced. Here the authors report a Korean reference genome and consensus variome, and show that an ethnically-relevant reference can improve variant detection.

## Graphical abstract

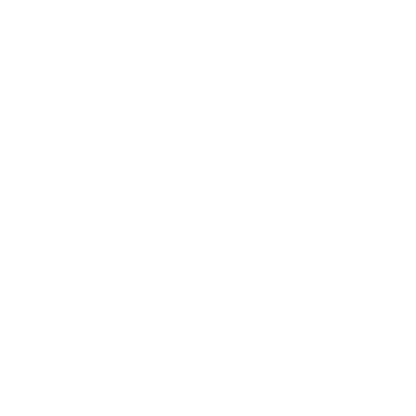

The standard human reference (currently GRCh38), which is mostly based on Caucasian and African ancestry^1^,^2^, is accurate, precise and extensive. Because of the relatively small long-term effective population size of anatomically modern humans (estimated to be as small as ∼10,000)^3^,^4^, such a reference is adequate for most purposes and routinely used in research and biomedical applications. However, certain population-specific variants could be missed with such a universal reference, and the current research efforts to map human diversity, including low frequency and structural variants, would benefit from ethnically relevant references^5^,^6^. Since the publication of the first draft of the human reference genome in 2001 (ref. 7), sequencing technologies have advanced rapidly. In 2007, the diploid genome of a Caucasian male was sequenced and assembled using Sanger sequencing technology (HuRef)^8^. Later, the genomes of a Chinese (YH), an African (2009), a Caucasian (HsapALLPATHS1, here called NA12878_Allpaths, 2011) and a Mongolian (2014) were built using Illumina short-read sequencing data^9^,^10^,^11^. In 2014, a complete hydatidiform mole genome (CHM1_1.1) was assembled, albeit reference-guided, using Illumina short reads and indexed bacterial artificial chromosome (BAC) clones^12^. In 2015, a haplotype-resolved diploid YH genome was assembled using fosmid pooling together with short-read sequence data^13^. These assemblies, although useful and important for genomics researches, are not of sufficient accuracy or overall quality to be considered a general purpose standard reference genome^14^.

The recent increased availability of long-range sequencing and mapping methods has important implications for the generation of references for ethnic groups and even personal genomes, especially for disease-associated structural variations (SVs). Long -range data can improve draft genome assemblies by increasing the scaffold size, efficiently closing gaps, resolving complex regions and identifying SVs^15^,^16^,^17^,^18^,^19^,^20^,^21^,^22^ at relatively low costs. Notable approaches are single-molecule real-time sequencing technology (SMRT) and highly parallel library preparation and local assembly of short reads (synthetic long reads) for resolving complex DNA regions and filling genomic gaps^15^,^16^,^17^. For instance, single haplotype human genomes were constructed using single-molecule long-read sequencing (CHM1_PacBio_r2 and CHM13). Long-read methods can be complemented and validated by two high-throughput mapping methods: optical mapping and nanochannel-based genome mapping. The most representative cases are the NA12878 (ASM101398v1; here called NA12878_single) and HX1 (a Chinese individual) genomes, which were hybrid assembled by combining single-molecule long reads with single-molecule genome maps^21^,^22^. Assemblies incorporating high-throughput short reads and long-range mapping or sequencing data, or hybrid assemblies, can enhance the quality, providing much longer scaffolds with validation and adjustment of complex genomic regions^19^,^20^,^21^,^22^.

Complementary to reference genome projects, which provide accurate templates, population genome projects, such as Personal Genome Project (PGP)^23^ and the 1,000 Genomes Project (1KGP)^24^,^25^, provide valuable variome information that is fundamental to many biomedical research projects. The PGP was initiated in 2005 to publicly share personal genome, health and trait data, crucial in understanding the diverse functional consequences associated with genetic variation. Recently, large-scale population genome projects in Britain and the Netherlands have been launched to identify population-specific rare genetic variations and disease-causing variants^26^,^27^. The single reference and population derived genomic variation types and frequencies (variome) are the pillars of genomics.

Here, we report two versions of the Korean reference (KOREF) genome (KOREF_S: a single reference assembly and KOREF_C: single reference+consensus variome), produced as part of PGP, by utilizing hybrid sequencing and mapping data. KOREF provides another high quality East-Asian reference to complement GRCh38. KOREF was initiated by the Korean Ministry of Science and Technology in 2006 to generate a national genome and variome references. To deal with the issues inherent to short reads, we use data from a number of different technologies (short and long paired-end sequences, synthetic and single-molecule long reads, and optical and nanochannel genome maps) to build a high quality hybrid assembly of a male donor, KOREF_S (Fig. 1). Furthermore, we integrate information from 40 high-coverage whole genomes (based on short reads) from the Korean PGP (KPGP)^28^ to generate a population-wide consensus Korean reference, KOREF_C. We compare the genomic structure of KOREF_C with other human genome assemblies, uncovering many structural differences, including ethnic-specific highly frequent structural variants. Importantly, the identification of SVs is largely affected by the sequencing platform used and assembly quality, suggesting the need for long-read sequences and a higher quality assembly to comprehensively map the ethnic and personal genomic structures. Accompanied by multi-ethnic PGP data, in the future, many low-cost personal, national and ethnic genome references will accelerate the completion of mapping all human genome diversity in both single-nucleotide variations (SNVs) and SVs.

## Results

### Choosing a representative genome donor

We recruited 16 Korean volunteers, who signed an informed consent (based on the PGP protocol, with minor country-specific adaptations) for use of their genomic data and agreed to their public release. After extracting DNA from peripheral blood (Supplementary Table 1), we genotyped each volunteer using an Infinium Omni1 quad chip. Multidimensional scaling plots of pairwise genetic distances were constructed, using an additional 34 Korean whole-genome sequences from the KPGP database, as well as 86 Japanese, 84 Chinese, 112 Caucasians and 113 Africans genotype data from HAPMAP phase 3 (ref. 29; Supplementary Fig. 1). All 16 Korean samples fell into a tight population cluster, indicating they represent one ethnic group. A healthy male donor was chosen as KOREF_S by considering a list of parameters such as centrality of the genetic distance, the participant’s age, parental sample availability, the availability for continuous blood sample donation and normality of the G-banded karyotype (Supplementary Fig. 2). To supply reference material, an immortalized cell line was constructed from the KOREF_S donor’s blood and deposited in the Korean Cell Line Bank (KCLB, #60211).

### KOREF_S assembly

We obtained short-read sequencing data from the Illumina HiSeq2000 and HiSeq2500 platforms, using the same approach adopted by other draft reference genome projects^9^,^10^,^11^,^13^,^30^. A total 964 Gb of paired-end DNA reads were generated from 24 libraries with different fragment sizes (170, 500 and 700 bp of short insert size, and 2, 5, 10, 15 and 20 Kb of long insert size), giving a total sequencing depth coverage of ∼311 fold (Supplementary Tables 2 and 3). From a *K*-mer analysis, the size of KOREF_S was estimated to be ∼3.03 Gb (Supplementary Table 4). A total of 68,170 scaffolds (≥200 bp) were generated, totalling 2.92 Gb in length reaching an N50 length of almost 20 Mb (19.85 Mb) and containing only 1.65% gaps (Table 1 and Supplementary Fig. 3). Approximately, 90% of the genome draft (N90) was covered by 178 scaffolds, each larger than 3.09 Mb, with the largest spanning over 80 Mb (81.9) on Chromosome 6.

To further extend the scaffolds, we used a high-throughput whole-genome optical mapping instrument, as previously suggested^18^. We extracted high molecular weight DNA and generated 745.5 Gb of single-molecule restriction maps (about two million molecules with 360 Kb of average size) from 67 high density MapCards, resulting in 240-fold optical map coverage (Supplementary Tables 5 and 6). To join the scaffolds, the single-molecule optical maps were compared to the assembled scaffolds that were converted into restriction maps by *in silico* restriction enzyme digestion. As a result, a total of 67 scaffolds (>200 Kb) were joined (Supplementary Table 7). This resulted in the increase of scaffold N50 length from 19.85 to 25.93 Mb (Table 1). Additionally, we generated two types of long reads for KOREF_S: PacBio SMRT (∼31.1 Gb, ∼10-fold coverage; Supplementary Fig. 4 and Supplementary Table 8) and Illumina TruSeq Synthetic Long Reads (TSLR, ∼16.3 Gb, ∼5.3-fold coverage; Supplementary Fig. 5 and Supplementary Table 9). Both types were used simultaneously, resulting in a decrease number of gaps from 1.75 to 1.06% of the expected genome size and a small increase in the final scaffold N50 length from 25.93 to 26.08 Mb (Table 1). We suspect that the low quantity of long reads (only 1.2% of read numbers compared with mate-pairs) is one reason for the small increase in the scaffold length (Supplementary Table 10).

Scaffolds usually contain misassembles^14^,^16^. We carefully and systematically assessed the quality of KOREF_S by generating nanochannel-based genome mapping data (∼145 Gb of single-molecule maps >150 Kb). We assembled the mapping data into 2.8 Gb of genome maps having an N50 length of 1.12 Mb (Supplementary Table 11). A total of 93.1% of KOREF_S scaffold regions (≥10 Kb) were covered by these genome maps, confirming their continuity (Supplementary Fig. 6). To pinpoint misassembles of KOREF_S scaffolds, we manually checked all the alignment results of the genome maps (3,216 cases with align confidence ≥20) onto KOREF_S and GRCh38. Seven misassembled regions were detected in KOREF_S and were split for correction (Supplementary Fig. 6). Next, we conducted a whole-genome alignment of KOREF_S and GRCh38 to detect possible inter- or intra-chromosomal translocations (indicative of misassembled sequences). A total of 280 of the KOREF_S scaffolds (≥10 Kb) covered 93.5% of GRCh38’s chromosomal sequences (non-gaps). We found no large-scale inter- or intra-chromosomal translocations. Additionally, as a fine-scale assessment, we aligned the short and long-read sequence data to the KOREF_S scaffolds (self-to-self alignment). A total of 98.85% of the scaffold sequences (>2 Kb) were covered by more than 20-fold. We assigned KOREF_S’s scaffolds to chromosomes using whole-genome alignment information (chromosomal location and ordering information of scaffolds on GRCh38 chromosomes), to obtain KOREF_S chromosome sequences (∼3.12 Gb of total length; Table 1).

### KOREF_C construction and genome annotation

Recently, Dewey *et al*. demonstrated much improved genotype accuracy for disease-associated variant loci using major allele reference sequences^5^, which were built by substituting the ethnicity-specific major allele (single base substitutions from the 1KGP) in the low-coverage European, African and East-Asian reference genomes. We followed the same approach for KOREF_S by substituting sequences with both SNVs and small insertions or deletions (indels) that were commonly found in the 40 KPGP high-depth (average 31-fold mapped reads) whole genomes. This removed individual-specific biases, and thus better represents common variants in the Korean population as a consensus reference (KOREF_C; Supplementary Table 12). Roughly two million variants (1,951,986 SNVs and 219,728 indels), commonly found in the 40 high quality short read Korean genome data, were integrated. Additionally, KOREF_S’s mitochondrial DNA (mtDNA) was independently sequenced and assembled, resulting in a 16,570 bp mitogenome that was similar, in structure, to that of GRCh38. A total of 34 positions of KOREF_S mtDNA were different from that of GRCh38 (Supplementary Table 13). KOREF_S’s mtDNA could be assigned to the D4e haplogroup that is common in East-Asians, whereas GRCh38 mtDNA belongs to European haplogroup H.

KOREF_C GC content and distribution were similar to other human assemblies except the African assembly, which has the lowest quality among them (Supplementary Fig. 7). We annotated KOREF_C for repetitive elements by integrating *de novo* prediction and homology-based alignments. Repetitive elements occupied 1.51 Gb (47.13%) of KOREF_C (Supplementary Table 14), which is slightly less than found in GRCh38 (1.59 Gb). On the other hand, KOREF_C contained more repeats than the Mongolian genome (1.36 Gb), which was assembled by next-generation sequencing short reads only. We predicted 20,400 protein-coding genes for KOREF_C (Supplementary Table 15 and ‘Methods’ section). By comparing KOREF_C with other human assemblies (GRCh38, CHM1_1.1, HuRef, African, Mongolian, and YH), a total of 875.8 Kb KOREF_C sequences (≥100 bp of fragments) were defined as novel (Supplementary Table 16 and Methods section).

### KOREF_C compared with other human genomes

We assessed the quality of nine publicly available human genome assemblies (CHM1_PacBio_r2, CHM1_1.1, NA12878_single, NA12878_Allpaths, HuRef, Mongolian, YH_2.0, African and KOREF_C) by comparing assembly statistics, and the recovery rates for GRCh38 genome, segmentally duplicated regions, and repetitive sequences (Table 2, Supplementary Tables 17–19). The results showed that KOREF_C was more contiguous (26.46 Mb of N50) than any of the short-read based *de novo* assemblies, but comparable to long-read based assemblies (26.83 Mb of N50 for NA12878_single; 26.90 Mb of N50 for CHM1_PacBio_r2); KOREF_C was hybrid assembled by compiling heterogeneous sequencing and mapping technologies, however, a majority of KOREF_C sequences was derived from next-generation sequencing short reads. However, KOREF_C’s contig size is small (47.86 Kb of N50 and 17,749 of L50; Supplementary Table 17) compared with long-read based assemblies due to the low level of continuity information of short reads. KOREF_C showed a comparable GRCh38 recovery rate with other long-read assemblies (Table 2 and Supplementary Table 18). KOREF (KOREF_S scaffolds) recovered duplicated and repetitive regions more efficiently than other short-read based *de novo* assemblies but less than the two PacBio long-read assemblies (Supplementary Table 19). Especially, the higher depth long-read assembly CHM1_PacBio_r2 recovered the most segmentally duplicated regions, almost as well as GRCh38, indicating that long-read information is important to recover such challenging genomic regions. Also, structural polymorphisms between the two haplotypes in a donor is one of the most significant factors affecting assembly quality^15^,^31^. Therefore, it was expected that CHM1_PacBio_r2, a haploid assembly, showed a superior genome recovery for segmentally duplicated regions than other assemblies using a diploid source. Additionally, we compared assembly quality by mapping the re-sequencing data of a single haplotype genome (CHM1) to the human assemblies (Supplementary Fig. 8). Ideally, CHM1 should lack heterozygous variants, if the human assembly recovered the entire genome well. CHM1_PacBio_r2 was the most accurate (having the lowest number of heterozygous variants) in resolving the entire human genome, and KOREF_C was the most accurate among the short-read based assemblies. These results confirm that short reads based *de novo* assemblies have reduced power to fully resolving the entire genome sequences accurately^14^.

We also conducted gene content assessments by comparing the number of detected RefSeq (ref. 32) protein-coding genes in each human assembly (Table 2 and Supplementary Table 20). The RefSeq genes were the best recovered in CHM1_1.1 (18,040), which was assembled using that reference as a guide. Among the *de novo* assembled genomes, KOREF_C contained the largest number (17,758) of intact RefSeq genes, even more than long-read based assemblies (∼17,657). Notably, the NA12878_single genome, which was hybrid assembled by combining single-molecule long reads with genome maps, had the lowest number (6,610) of intact protein-coding genes, even lower than the low quality African genome (9,167). We confirmed that NA12878_single had many frame-shifts in its coding regions. This can be explained by the higher error rates of PacBio single-molecule long reads, which could not be corrected by an error correction step due to its low sequencing depth (46 × coverage)^21^,^33^.

### Structural variation comparison

We investigated SVs, such as large insertions, deletions and inversions, in these eight human assemblies by comparing to GRCh38 (since there were no paired-end read data, HuRef was not used in this analysis). Our analysis showed that assembly quality is determined primarily by sequencing platform (that is, sequence read lengths), and therefore, we had to consider that mis-assemblies could generate erroneous SVs. Two Caucasian samples (CHM1 and NA12878) were assembled using short-read sequences as well as long reads, and therefore, allow an examination of the association between the assembly quality and SV identification. The CHM1 sample’s ethnicity was confirmed to be Caucasian using ancestry-sensitive DNA markers in autosomes^34^ and mitochondrial DNA sequences (Supplementary Fig. 9). SVs that could be derived from possible misassembles were filtered out by comparing the ratio of aligned single-end reads to paired-end reads (S/P ratio) as previously suggested^35^ (see the ‘Methods’ section).

A total of 6,397 insertions (>50 bp), 3,399 deletions (>50 bp) and 42 inversions were found in KOREF_C compared with GRCh38, for a total of 9,838 SVs. This is slightly fewer than found in the Mongolian (12,830 SVs) and African (10,772 SVs) assemblies, but greater than those found in CHM1 and NA12878 assemblies (∼5,179 SVs; Table 3, Supplementary Tables 21 and 22). Notably, YH_2.0 (5,027 SVs) had a similar number of SVs to those found in the Caucasian assemblies, compared with other Asian assemblies. The length distribution of SVs found in these assemblies showed a similar pattern (Supplementary Figs 10 and 11), with a peak at the 200–400 bp size range, due to *Alu* element insertions and deletions^15^,^35^. The fractions of SVs in repeat regions were higher in the short-read based assemblies (69.6–81.9%) than long-read assemblies (67.7–68.7%; Table 3 and Supplementary Table 23). On the other hand, the fractions of SVs in the segmentally duplicated regions were much higher in the long-read assemblies (21.4–29.0%) than short-read assemblies (3.9–12.6%; Table 3 and Supplementary Table 24).

**Figure 2:**
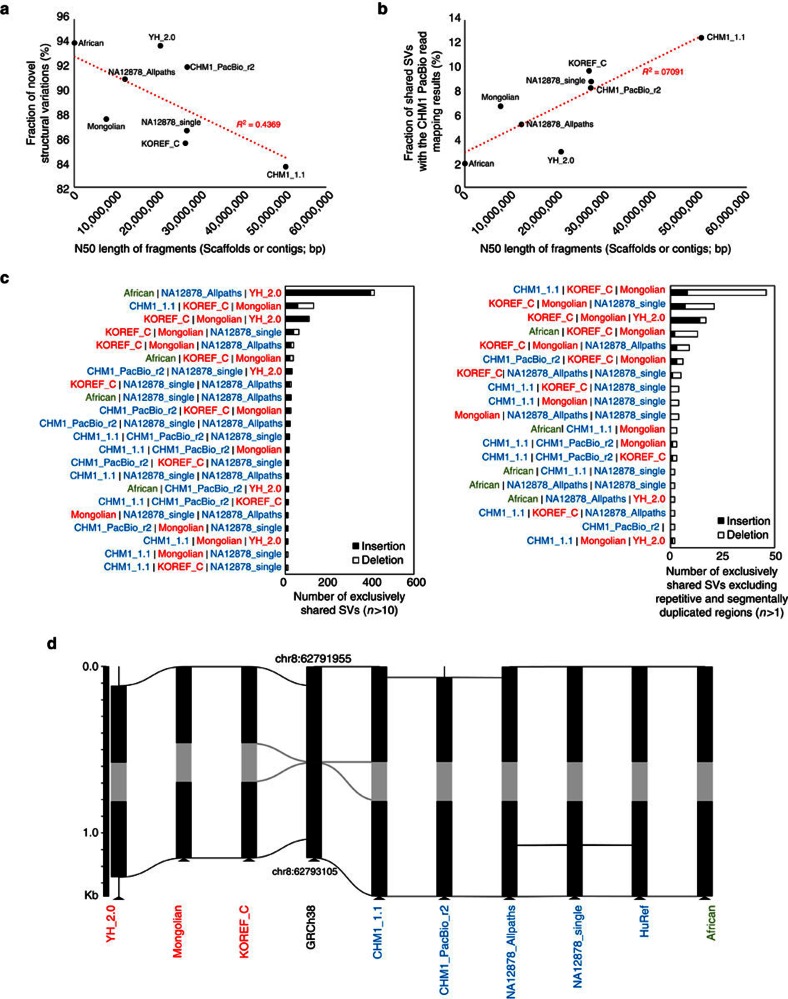
SVs among human assemblies. (**a**) The correlation between N50 length of fragments (scaffolds or contigs) and fraction of novel SVs. (**b**) The correlation between N50 length of fragments and fraction of SVs shared with the CHM1 PacBio read mapping method. (**c**) Exclusively shared SVs among human assembly sets. SVs shared (reciprocally 50% covered) by only denoted assemblies were considered in this figure. (**d**) An example of SV that was shared by nine human assemblies. Grey regions denote structural differences shared among all the assemblies, and horizontal lines indicate homologous sequence regions.

Of the KOREF_C SVs 93.8% of insertions and 70.4% of deletions were not found in public SV databases and hence defined as novel (Table 3, Supplementary Fig. 10, Supplementary Table 25 and ‘Methods’ section). The fraction of novel SVs in KOREF_C was similar to those found in other human assemblies but smaller than other short-read only *de novo* assemblies. Regardless of sequencing platform, all assemblies showed a greater fractions of novel SVs than those found by mapping CHM1’s PacBio SMRT reads to the human reference genome (here termed CHM1_mapping)^15^. Notably, CHM1_PacBio_r2, which was assembled using the same sample’s PacBio long reads, also showed a much higher fraction of novel SVs. We found a correlation between N50 length of fragments and the fraction of novel SVs (*R*^2^=0.44; Fig. 2a). When we compared SVs of the human assemblies with the SVs by the CHM1_mapping, only small portions of SVs (∼12.51%) were shared (Table 3 and Supplementary Table 26). The shared portion of SVs (8.85%) between the CHM1_PacBio_r2 and CHM1_mapping was small, and the shared portions of NA12878 assemblies were quite different (NA12878_single: 8.32%, NA12878_Allpaths: 5.27%). There was a correlation between the assembly quality (N50 length) and shared portion (*R*^2^=0.71; Fig. 2b). These results suggest that even for the same sample there was a large difference between the long-read mapping and *de novo* assembly based whole-genome alignment methods.

Human genomes contain population-specific sequences and population stratified copy number variable regions^6^,^36^. Therefore, we assumed that ethnically relevant human assemblies should share similar genome structures. To investigate the genomic structure among human assemblies, we grouped SVs that were shared by the human assemblies (Fig. 2c). Most SVs (above 61.6%) were assembly specific (Supplementary Table 27). When we consider SVs that were shared by only two assemblies, two Asian genomes (KOREF_C and Mongolian) shared the highest number of SVs (Supplementary Fig. 12). However, YH_2.0 shared only small numbers of SVs with KOREF_C and Mongolian assemblies. Notably, YH_2.0 and African genomes shared SVs abundantly, which cannot be explained by our assumption that similar ethnic genomes should have a higher genome structure similarity. CHM1_PacBio_r2 and NA12878_single, which are Caucasian assemblies using PacBio long-read sequences, shared more SVs than those between the same sample’s assemblies (NA12878 assemblies and CHM1 assemblies). In cases of SVs shared by only three assemblies, African, NA12878_Allpaths, and YH_2.0 had the largest number of shared SVs, whereas the three Asian genomes had smaller numbers of shared SVs (Fig. 2c and Supplementary Fig. 12). However, when SVs detected in the repetitive and segmentally duplicated regions were excluded, the three Asian assemblies had the largest number of shared insertions, whereas African, NA12878_Allpaths and YH_2.0 shared no insertions at all (Supplementary Fig. 13). These results indicate that SV identification was critically affected by the sequencing platform and assembly quality. We therefore suggest that long-read sequencing methods are necessary to improve the assembly quality and SV identification for the better characterization of genome structural differences.

Given these limitations, we continued to identify commonly shared SVs by ethnic group. To do this, we checked S/P ratios for the SVs using the whole-genome re-sequencing data from five Koreans, four East-Asians, four Caucasians and one African, from the KPGP, 1KGP, Human Genome Diversity Project (HGDP)^37^, and the Pan-Asian Population Genomics Initiative (PAPGI). First, we found one SV that was shared by all human assemblies (Fig. 2d). This SV was also commonly found in re-sequencing data (13 out of the 14 re-sequencing data). Out of the 110 SVs that were shared by the three Asian assemblies, 18 were frequently found in eleven Asian genomes (one Mongolian assembly, one Chinese assembly and nine Asian re-sequencing data) compared with 10 non-Asian genomes (five non-Asian assemblies and five re-sequencing data, *P* value <0.05, Fisher’s exact test; Supplementary Table 28). Although the SV analysis had limitations due to the heterogeneity of sequencing platform and assembly quality, these results may indicate that the genomic structure is more similar within the same ethnic group^6^,^36^, suggesting that ethnically relevant reference genomes are necessary for efficiently performing large-scale comparative genomics.

### Variant comparison mapped to KOREFs

**Figure 3:**
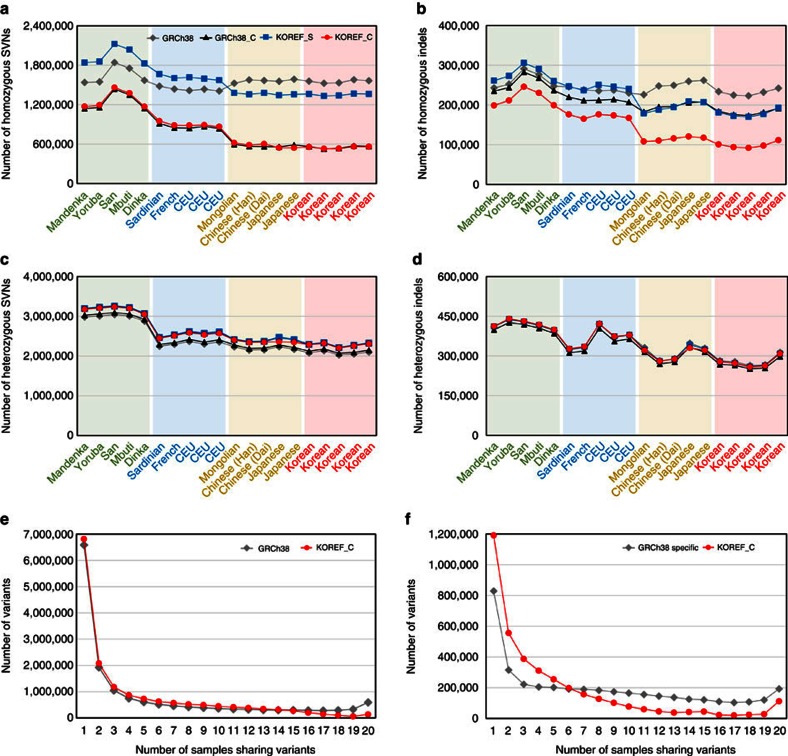
Variants difference depending on the reference genome. Variants (SNVs and small indels) numbers within the regions shared by KOREFs, GRCh38 and GRCh38_C were compared using whole-genome re-sequencing data from three different ethnic groups (Africans: Mandenka, Yoruba, San, Mbuti and Dinka; Caucasians: Sardinian, French and three CEPH/Utah (CEU); East-Asians: Mongolian, two Chinese, two Japanese and five Koreans). (**a**) Number of homozygous SNVs. (**b**) Number of homozygous small indels. (**c**) Number of heterozygous SNVs. (**d**) Number of heterozygous small indels. (**e**) The number of variants (referenced by GRCh38 and KOREF_C) at different levels of sharedness. (**f**) The number of reference-specific variants at different levels of sharedness.

Ethnicity-specific genomic sequences that are absent from the reference genome may be important for precise detection of genomic variations^22^. It is also known that the current human reference sequence contains both common and rare disease risk variants^38^, and the use of the current human reference for variant identification may complicate the detection of rare disease risk alleles^5^. Using re-sequencing data on five whole genomes from each population (Caucasian, African, East-Asian and Korean), we compared the number of variants (SNVs and small indels) detected using KOREF_S, KOREF_C, GRCh38, and consensus Asian GRCh38 (GRCh38_C, the implementation of Dewey *et al*.’s Asian major allele reference^5^ but including small indels for our study; Supplementary Tables 29 and 30). We found that the number of variants was considerably different, depending on the reference used. Variant numbers of all individuals (Caucasian, African and East-Asian) decreased when KOREF_C was used as a reference. However, because the lower number of actual bases (non-gapped) in KOREFs (KOREF_S and KOREF_C) could affect the accuracy of genotype reconstruction, we compared variant numbers only within the regions shared by KOREFs, GRCh38 and GRCh38_C (Supplementary Table 31). As expected, the numbers of homozygous variants from all the Asian genomes (two Chinese, two Japanese, one Mongolian and five Korean) decreased largely (35.5% of SNVs and 43.9% of indels remained) when KOREF_C was used as a reference compared with GRCh38 (Fig. 3a,b); on the contrary, the numbers of homozygous variants from Caucasian and African genomes decreased little. In cases of homozygous SNVs, a similar pattern was observed between GRCh38_C and KOREF_C. However, the numbers of homozygous indels when using GRCh38_C as a reference were higher than when using KOREF_C as a reference. We speculate that this is because fewer common indels were substituted for GRCh38_C when compared with KOREF_C due to low sequencing depths of 1KGP data. The numbers of homozygous variants found in non-Korean Asians were similar to those found among Koreans, suggesting that KOREFs can be used for other East-Asian genomes. On the other hand, the numbers of heterozygous SNVs were slightly higher in KOREFs, which is consistent with the mapping result of the CHM1 re-sequencing data as described above (Supplementary Fig. 8). However, we confirmed that the numbers of heterozygous SNVs were similar when restricted our analysis to non-repetitive regions. The numbers of heterozygous indels were also largely constant regardless of reference used (Fig. 3c,d).

Focusing on differently called variants (variants found in GRCh38 but not found in KOREF_C, and vice versa), we found that there were differences in the number of variants among populations (that is, population stratification in terms of variant number). The differences of variants among populations were more prominent when using KOREF_C specifically called variants (Supplementary Table 32). The number of commonly shared KOREF_C called variants (>6 individuals) in the 20 whole genomes was much smaller, whereas the number of less common KOREF_C called variants, including individual-specific ones, was higher (Fig. 3e,f). Also, the number of KOREF_C specifically called variants was considerably lower in the 10 Asians than those in the 10 non-Asians. These results reflect the consensus variants components of KOREF_C and also confirm that GRCh38 lacks Asian specific sequences^5^. The majority (92.3%) of the GRCh38 specifically called variants were found in single-nucleotide polymorphism database (dbSNP)^39^ (Supplementary Table 32), whereas a smaller fraction (56.17%) of the KOREF_C specifically called variants were defined as known. When variants in repetitive and segmentally duplicated regions were excluded, a much larger fraction (86.21%) of the KOREF_C specifically called variants were known (Supplementary Table 33), indicating that the majority of novel variants found in KOREF_C was caused by the incompleteness of repetitive and segmentally duplicated regions. Therefore, we conclude that although KOREFs have an advantage for efficient variant detection for the same ethnic genomes, KOREFs need to be improved using longer sequence reads to reconstruct genotypes properly.

Additionally, we found that the number of variants identified following substitution in the reference with the dominant variant (KOREF_S versus KOREF_C) is much higher than the change caused by the ethnicity difference (KOREF_S versus GRCh38; Fig. 3a,b). Also, the East-Asians’ homozygous variant number decreased only slightly when the KOREF_S was used, compared with GRCh38 (87.0% of homozygous SNVs and 77.9% of homozygous indels remained), while it was greatly decreased when KOREF_C was used (36.1% of homozygous SNVs and 44.5% of homozygous indels remained). On the other hand, the number of non-East Asians’ homozygous variants increased when the KOREF_S was used, compared with when GRCh38 was used. These results indicate that, at the whole-genome variation level, intra-population variation is higher than the inter-population variation in terms of number of variants, supporting the notion that *Homo sapiens* is one population with no genomically significant subspecies.

### Ethnicity-specific reference and functional markers

We also found that depending on the reference used, different numbers of non-synonymous SNVs (nsSNVs) and small indels were found in genic regions (Supplementary Tables 34 and 35). With the aforementioned ten East-Asian whole genomes, the number of homozygous nsSNVs (from 3,644 to 1,280 on average) and indels (from 95 to 40 on average) decreased most when using KOREF_C as a reference instead of GRCh38; whereas a smaller decrease was observed in the five Caucasians (nsSNVs from 3,467 to 2,098; indels from 89 to 65) and five Africans (nsSNVs from 4,216 to 3,007; indels from 134 to 109). When KOREF_C was used as the reference, predicted functionally altered (or damaged) genes by the homozygous variants also decreased the most among the East-Asians (East Asians, from 490 to 246 on average; Caucasians, from 448 to 362; Africans, from 448 to 415; Supplementary Table 36). Notably, in the 10 East-Asians, the functionally altered genes, which were found only against GRCh38 but not KOREF_C, were enriched in several disease terms (myocardial infarction, hypertension and genetic predisposition to disease), and olfactory and taste transduction pathways (Supplementary Tables 37 and 38). Additionally, 13 nsSNVs, which are known as disease- and phenotype-associated variants, were called against GRCh38 but not KOREF_C (Supplementary Table 39); we verified these loci by manually checking short reads alignment to both GRCh38 and KOREF_C (Supplementary Fig. 14).

## Discussion

In the era of large-scale population genome projects, leveraging ethnicity-specific reference genomes alongside GRCh38 could bring additional benefits in detecting variants. This is because each ethnic group has a specific variation repertoire, including single-nucleotide polymorphisms and larger structural deviations^6^,^40^. Population stratification (systematic difference in allele frequencies) can be a problem for association studies, where the association could be found due to the underlying structure of the population and not a disease-associated locus^41^. Ethnicity-specific genomic regions such as novel sequences and copy number variable regions can affect precise genotype reconstruction. We demonstrate an example of a better genotype reconstruction in the copy number variable regions using KOREF (Supplementary Fig. 15). Hence, our ethnicity-specific reference genome, KOREF, may also be useful for detecting disease-relevant variants in East-Asians.

*De novo* assembly based on Sanger sequencing is still too expensive to be used routinely. We have demonstrated that it is possible to produce a *de novo* assembly of relatively high quality at a fraction of the cost by combining the latest sequencing and bioinformatics methods. Additionally, we have shown that optical and nano technologies can extend the size of the large scaffolds while validating the initial assembly. We found that the identification of structural differences based on the genome assembly is largely affected by assembly quality, suggesting a need for new technologies and higher quality of assembly from additional individuals in various populations to better understand comprehensive maps of genomic structure. Also, it is important that the same coordinate system on the GRCh38 allows comparison of different individuals, to leverage the vast amount of previously established knowledge and annotations. Therefore, it is also crucial to investigate how to transfer those annotations to personal or ethnic reference genomes by preferentially supplementing additional references into GRCh38 to gain additional biological insights. KOREFs cannot, and are not meant to, replace the human reference, and some of its genomic regions, such as centromeric and telomeric regions, and many gaps, are largely incomplete. However, KOREFs still can be useful in improving the alignment of East-Asian personal genomes, in terms of fast and efficient variant-calling and detecting individual- and ethnic-specific variations for large-scale genome projects.

## Methods

### Sample preparation

All sample donors in this study signed written informed consent to participate, and the Institutional Review Board on Genome Research Foundation (IRB-201307-1 and IRB-201501-1 for KOREF and 20101202-001 for KPGP) provided approval for this study. Genomic DNA and RNA used for genotyping, sequencing, and mapping data were extracted from the peripheral blood of sample donors. We conducted genotyping experiments with 16 Korean male participants using Infinium Omni1 quad chip to check if the 16 donors had certain genetic biases. A total of 45 Korean whole genomes (40 for variant substitution and five for variant comparison) were used in this study (from the KPGP), sequenced using Illumina HiSeq2000/2500. For the comparison with the 16 donors, 34 Korean whole-genome sequences from the KPGP and 86 Japanese, 84 Chinese, 112 Caucasians and 113 Africans genotyping data from HAPMAP phase 3 were used. After filtering for MAF (<5%), genotyping rate (<1%), and LD (*R*^2^≤0.2) using PLINK^42^, 90,462 and 72,578 shared nucleotide positions were used to calculate genetic distances for three ethnic groups (East-Asians, Caucasians and Africans) and three East-Asian groups (Koreans, Chinese and Japanese), respectively.

Epstein–Barr virus (EBV)-transformed B-cell line was constructed from the KOREF_S donor’s blood^43^, with minor modification. Briefly, peripheral blood mononuclear cells were purified by Ficoll-Paque Plus (GE Healthcare, UK) density gradient centrifugation. For EBV infection, the cells were pre-incubated for 1 h with spent supernatant from the EBV producer cell line B95-8, and then cultured in RPMI-1640 containing 10–20% foetal bovine serum, 2 mM L-glutamine, 100 U ml^−1^ penicillin, 0.1 mg ml^−1^ streptomycin, 0.25 μg ml^−1^ amphotericin B (all from Gibco, Grand Island, NY, USA). The EBV-transformed B-cells were maintained at a concentration between 4 × 10^5^–1 × 10^6^ cells ml^−1^ and expanded as needed.

### Genome sequencing and scaffold assembly

For the *de novo* assembly of KOREF_S, 24 DNA libraries (three libraries for each insert size) with multiple insert sizes (170 bp, 500 bp, 700 bp, 2 Kb, 5 Kb, 10 Kb, 15 Kb and 20 Kb) were constructed according to the protocol of Illumina sample preparation. The libraries were sequenced using HiSeq2500 (three 20 Kb libraries) and HiSeq2000 (others) with a read length of 100 bp. PCR duplicated, sequencing and junction adaptor contaminated, and low quality (<Q20) reads were filtered out, leaving only high accurate reads to assemble the Korean genome. Additionally, short insert size and long insert size reads were trimmed into 90 bp and 49 bp, respectively, to remove poly-A tails and low quality sequences in both ends. Error corrected read pairs by *K*-mer analysis from the short insert size libraries (<1 Kb) were assembled into distinct contigs based on the *K*-mer information using SOAPdenovo2 (ref. 30). Then, read pairs from all the libraries were used to concatenate the contigs into scaffolds step by step from short insert size to long insert size libraries using the *scaff* command of SOAPdenovo2 with default options except the −F option (filling gaps in scaffolds). To obtain scaffolds with the longest N50 length, we assembled the Korean genome (KOREF_S) with various *K*-mer values (29, 39, 49, 55, 59, 63, 69, 75 and 79) and finally selected an assembly derived from *K*=55, which has longest contig N50 length. To reduce gaps in scaffolds, we closed the gaps twice using short insert size reads iteratively.

### Super-scaffold assembly

We used whole-genome optical mapping data to generate a restriction map of the KOREF_S and assemble scaffolds into super-scaffolds^18^. First, 13 restriction enzymes were evaluated for compatibility with the Korean genome draft assembly, and *SpeI* enzyme was deemed suitable for the Korean genome analysis. High molecular weight DNA was extracted, and 4,217,937 single-molecule restriction maps (62,954 molecules on each map card on overage) were generated from 67 high density MapCards. Among them, 2,071,951 molecules exceeding 250 Kb with ∼360 Kb of average size were collected for the genome assembly. The Genome Builder bioinformatics tool of OpGen^18^ was used to compare the optical mapping data to the scaffolds. The distance between restriction enzyme sites in the scaffolds were matched to the lengths of the optical fragments in the optical maps, and matched regions were linked into super-scaffolds. Only scaffolds exceeding 200 Kb were used in this step.

Additionally, we generated two types of long reads for KOREF_S building: PacBio long reads and TSLRs. The PacBio long reads were generated using a Pacific Biosciences RSII instrument (P4C2 chemistry, 78 SMRT cells; P5C3 chemistry and 51 SMRT cells), and the TSLRs were sequenced by Illumina HiSeq2500. Both long reads were simultaneously used in additional scaffolding and gap closing processes using PBJelly2 programme^44^ with default options.

### Assembly assessment and chromosome building

For a large-scale assessment of the scaffolds, we generated nanochannel-based genome mapping data (∼145 Gb of single-molecule maps exceeding 150 Kb) on five irysChips and assembled the mapping data into 2.8 Gb of consensus genome maps using BioNano Genomics Irys genome mapping system. The consensus genome maps were compared with KOREF_S scaffolds and GRCh38 using irysView software package^21^ (version 2.2.1.8025). To identify misassembles in KOREF_S scaffolds in detail, we manually checked alignment results of the consensus genome map into KOREF_S scaffolds and human reference. For a smaller resolution assessment, we aligned all the filtered short and long reads into the scaffolds using BWA-MEM^45^ (version 0.7.8) with default options. We conducted a whole-genome alignment between KOREF_S scaffolds (≥10 Kb) and human reference (soft repeat masked) using SyMap^46^ with default comparison parameters (mapped anchor number ≥7) to detect possible inter- or intra-chromosomal rearrangements. We manually checked all the whole-genome alignment results.

To build the chromosome sequence of KOREF_S, first we used the whole-genome alignment information (chromosomal location and ordering information) of the final scaffolds (≥10 Kb) onto GRCh38 chromosomes. Then, unmapped scaffolds were re-aligned to GRCh38 chromosome with a mapped anchor number ≥4 option. Small length scaffolds (from 200 bp to 10 Kb) were aligned to GRCh38 chromosomes using BLASR^47^, and only alignments with mapping quality=254 were used. Unused scaffolds (a total 88.3 Mb sequences) for this chromosome building process were located in an unplaced chromosome (chrUn). Gaps between the aligned scaffolds were estimated based on the length information of the human reference sequences. If some scaffold locations overlapped, 10 Kb was used as the size of gap between the scaffolds. We added 10 Kb gaps on both sides of KOREF_S chromosome sequences as telomeric regions just as done for GRCh38. The mitochondrial sequences of KOREF_S were independently sequenced using Nextera XT sample prep kit and then assembled using ABySS (ref. 48) (version 1.5.1) with *K*=64. Haplogroup of the mitochondrial DNA was assigned using MitoTool^49^.

The 40 Korean whole-genome sequences from KPGP database were aligned onto KOREF_S chromosomes using BWA-MEM with default options, to remove individual-specific sequence biases of KOREF_S and generate KOREF_C. SNVs and small indels in the 40 Koreans were called using the Genome Analysis Toolkit (GATK, version 2.3.9)^50^. IndelRealigner was conducted to enhance mapping quality, and base quality scores were recalibrated using the TableRecalibration algorithm of GATK. Commonly found variants in the 40 Korean genomes were used to substitute KOREF_S sequences. For the SNV substitution, we calculated allele ratio of each position, and then we substituted any KOREF_S sequence with the most frequent allele only if the KOREF_S sequence and most frequent allele were different. For the indel substitution, we used only indels that were found in over 40 haploids out of the 40 Korean whole genomes (80 haploids). In cases of sex chromosomes, we used 25 male (25 haploids) whole genomes for Y chromosome and 15 female whole genomes (30 haploids) for X chromosome comparison.

### Genome annotation

KOREF_C was annotated for repetitive elements and protein-coding genes. For the repetitive elements annotation, we searched KOREF_C for tandem repeats and transposable elements using Tandem Repeats Finder (version 4.07)^51^, Repbase (version 19.02)^52^, RepeatMasker (version 4.0.5)^53^ and RepeatModeler (version 1.0.7)^54^. For the protein-coding gene prediction, homology-based gene prediction was first conducted by searching nucleotides of protein-coding genes in Ensembl database 79 against KOREF_C using Megablast^55^ with identity 95 criterion. The matched sequences were clustered based on their positions in KOREF_C, and a gene model was predicted using Exonerate software^56^ (version 2.2.0). We also conducted *de novo* gene prediction. To certify expression of a predicted gene, we sequenced three different timeline whole transcriptome data of the KOREF_S sample using a TruSeq RNA sample preparation kit (v2) and HiSeq2500. We predicted protein-coding genes with the integrated transcriptome data using AUGUSTUS^57^ (version 3.0.3). We filtered out genes shorter than 50 amino acids and possible pseudogenes having stop-codons. We searched *de novo* predicted genes against primate (human, bonobo, chimpanzee, gorilla and orangutan) protein sequences from NCBI, and filtered out *de novo* predicted genes if identity and coverage were below 50%. For the assembly quality comparison purpose, we only used homology-based search for RefSeq (ref. 32) human protein-coding genes and repetitive elements. The homology-based segmental duplicated region search was conducted using DupMasker programme^58^. To calculate GRCh38 genome recovery rates of human assemblies, we conducted whole-genome alignments between each assembly (KOREF_S final contigs, KOREF_S final scaffolds and other assemblies) and GRCh38 using LASTZ^59^ (version 1.03.54) and Kent utilities (written by Jim Kent at UCSC)^60^ with GRCh38 self-alignment options (--step 19 --hspthresh 3000 --gappedthresh 3000 --seed=12of19 --minScore 3000 --linearGap medium). After generating a MAF file, we calculated genome recovery rates using mafPairCoverage in mafTools^61^.

To estimate the amount of novel KOREF_C sequences, we aligned the short insert size and long mate pair library sequences into GRCh38 using BWA-MEM with default options and then extracted unmapped reads using SAMtools^62^ (version 0.1.19) and Picard (version 1.114, http://picard.sourceforge.net) programs. We filtered out possible microbial contamination by searching against Ensembl databases of bacterial genomes and fungal genomes using BLAST with default options. The remaining reads were sequentially aligned into other human genome assemblies (CHM1_1.1, HuRef, African, Mongolian and YH sequentially) using BWA-MEM with default options, and then removed duplicated reads using MarkDuplicate programme in Picard. The alignment results were extracted to an unmapped BAM file using SAMtools view command with -u -f 4 options. We extracted final unmapped reads from the unmapped BAM file using SamToFastq programme in Picard. Finally, unmapped reads to the other human genome assemblies were aligned to KOREF_C. The regions with length ≥100 bp and covered by at least three unmapped reads were considered as novel in KOREF_C.

### Variant and genome comparison

A total of 15 whole-genome re-sequencing data results (five Caucasians, five Africans and five East-Asians) were downloaded from the 1KGP, HGDP and PAPGI projects. The re-sequencing data (five Caucasians, five Africans, five East-Asians and five Koreans from KPGP) was filtered (low quality with a Q20 criterion and PCR duplicated reads) and then mapped to KOREFs (KOREF_S and KOREF_C) with unplaced scaffolds, GRCh38, and GRCh38_C chromosomes using BWA-MEM with default options. To generate GRCh38_C, common variants (2,043,259 SNVs and 197,885 small indels) of East-Asians were collected from the 1KGP database and used to substitute GRCh38 sequences. The variants (SNVs and small indels) were called for only chromosome sequences using GATK, to exclude variants in unmatched and partially assembled repetitive regions^14^. Variants were annotated using SnpEff^63^, and biological function altering was predicted using PROVEAN^64^. We considered all of the nsSNVs causing stop codon changes and frame shift indels as function altered. Enrichment tests and annotation of variants were conducted using WebGestalt^65^ and ClinVar^66^. The variants were compared with dbSNP^39^ (version 144) to annotate known variants information.

For linking variants found compared with KOREFs, GRCh38 and GRCh38_C, the genome to genome alignment was conducted between GRCh38 and KOREF_C reference genomes using LASTZ^59^. The LASTZ scoring matrix used was with *M*=254 (--masking=254), *K*=4500 (--hspthresh=4,500), *L*=3,000 (--gappedthresh=3,000), *Y*=15,000 (--ydrop=15,000), H=0 (--inner=9), E=150/O=600 (--gap=<600,150>), and *T*=2 options. The LASTZ output was translated to the chain format with axtChain, then merged and sorted by the chainMerge and chainSort programs, respectively. The alignable regions were identified with chainNet, and then selected by netChainSubSet programs for creating a lift-over file. All programs run after LASTZ were written by Jim Kent at UCSC^60^.

To detect SVs among the human genome assemblies, we conducted whole-genome alignments between each assembly and GRCh38 using LASTZ. Then, the whole-genome alignment results were corrected and re-aligned based on a dynamic-programming algorithm using SOAPsv package. SVs that could be derived from possible misassembles were filtered out by comparing the S/P ratio for each SV region in the assembly and GRCh38; authentic SVs would be covered by sufficient paired-end reads, whereas spurious SVs would be covered by wrongly mapped single-end reads. We implemented the S/P ratio filtering system according to the previous published algorithm^35^, because the S/P ratio filtering step in the SOAPsv package is designed for only assembled sequences by SOAPdenovo. *P* value was calculated by performing Fisher’s exact test to test whether the S/P ratio of each SV and the S/P ratio of the whole genome are significantly different (*P* value<0.001). We confirmed that commonly shared SVs were not caused by the mis-assembly by checking the mapping status of KOREF_S short and long reads into both GRCh38 and KOREF_C. SVs by mapping CHM1’s PacBio SMRT reads to the human reference genome were derived by lift-over SV results found against GRCh37 in the published paper^15^. When we compared SVs in the different genome assemblies and available database, we considered SVs to be the same if SVs were reciprocally 50% covered and had the same SV type. Novel SVs were determined as not found in dbVar, Database of Genomic Variants (DGV)^67^, Database of Retrotransposon Insertion Polymorphisms (dbRIP)^68^, dbSNP146, Mills^69^, and 1000 Genome phase 3 database.

### Data availability

The Korean reference genome project has been deposited at DDBJ/ENA/GenBank under the accession LWKW00000000. The version described in this paper is version LWKW01000000. Raw DNA and RNA sequence reads for KOREF and KPGP have been submitted to the NCBI Sequence Read Archive database (SRA292482, SRA268892). The immortalized cell line of KOREF was deposited in the Korean Cell Line Bank (KCLB, #60211). All other data can be obtained from the authors upon reasonable request. All future KOREF updates will be available from www.koreanreference.org.

## Additional information

**How to cite this article:** Cho, Y. S. *et al*. An ethnically relevant consensus Korean reference genome is a step towards personal reference genomes. *Nat. Commun.* **7,** 13637 doi: 10.1038/ncomms13637 (2016).

**Publisher’s note**: Springer Nature remains neutral with regard to jurisdictional claims in published maps and institutional affiliations.

## Supplementary Material

Supplementary Information Supplementary Figures 1-15 and Supplementary Table 1-39

## Figures and Tables

**Figure 1:**
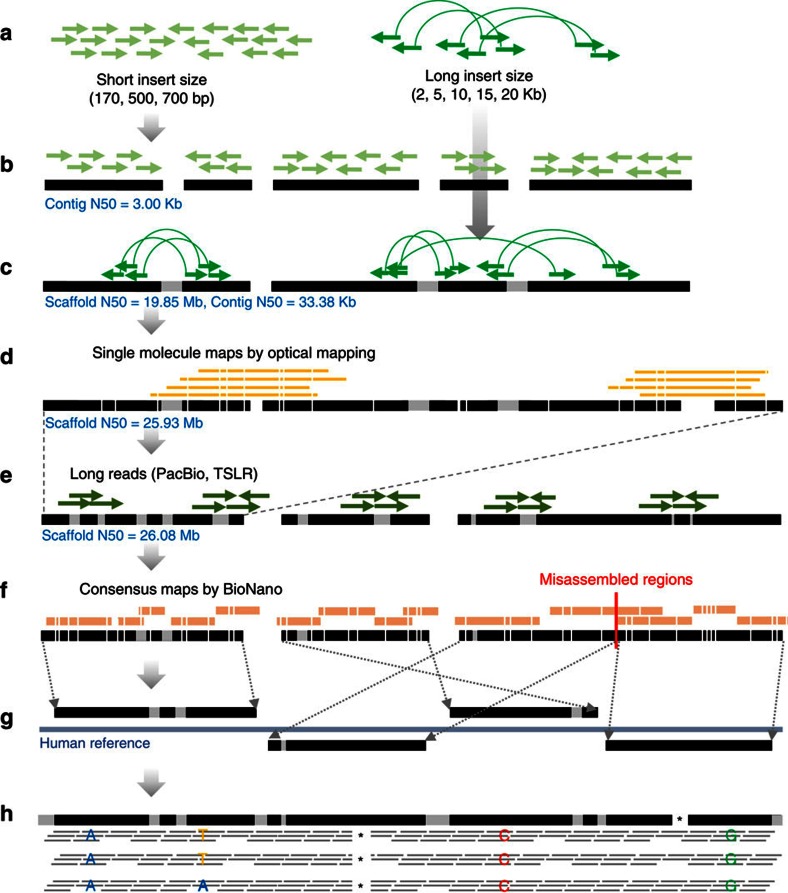
Schematic overview of KOREF assembly procedure. (**a**) Short and long insert size libraries by Illumina whole-genome sequencing strategy. (**b**) Contig assembly using *K*-mers from short insert size libraries. (**c**) Scaffold assembly using long insert size libraries. (**d**) Super-scaffold assembly using OpGen whole-genome mapping approach. (**e**) Gap closing using PacBio long reads and Illumina TSLR. (**f**) Assembly assessment using BioNano consensus maps. (**g**) Chromosome sequence building using whole-genome alignment information into the human reference (GRCh38). (**h**) Common variants substitution using 40 Korean whole-genome sequences.

**Table 1:** KOREF build statistics along the assembly steps.

	**Contig**		**Scaffold**		**Whole-genome optical mapping**		**Long reads (PacBio and TSLR)**		Chromosomes (assessment using BioNano maps)*	
	**Size (Kb)**	**No.**	**Size (Mb)**	**No.**	**Size (Mb)**	**No.**	**Size (Mb)**	**No.**	**Size (Mb)**	**No.**
N90	8.59	89,240	3.09	178	3.86	140	3.53	143	81.54	19
N80	14.62	63,987	6.45	116	9.45	92	9.26	93	103.05	16
N70	20.42	47,417	10.45	81	14.47	67	14.53	67	136.43	13
N60	26.58	35,099	16.16	59	19.56	49	19.36	50	137.59	11
N50	33.38	25,446	19.85	42	25.93	36	26.08	36	155.88	8
Longest	334.16	—	81.91	—	101.22	—	101.48	—	251.92	—
Gaps	0%	—	1.65%	—	1.75%	—	1.06%	—	9.44%	—
Total (≥200 bp)	2.87 Gb	230,514	2.92 Gb	68,170	2.92 Gb	68,103	2.94 Gb	68,451	3.12 Gb	24
Total (≥10 Kb)	2.52 Gb	82,254	2.88 Gb	1,243	2.88 Gb	1,176	2.90 Gb	1,369	3.12 Gb	24

*unplaced scaffolds were excluded.

**Table 2:** Systematic comparison of assembly quality.

**Assembly**	**Total sequence length (bp)**	**Scaffold or contig N50 (Mb)/L50**	**GRCh38 recovery rate (%)**	**Segmental duplication length (bp)**	**Repeat length (bp)**	**Detected RefSeq genes (intact only)**
GRCh38^C^	3,209,286,105	67.79/16	—	212,777,868 (6.63%)	1,564,209,365 (48.74%)	20,135
KOREF_C^S,L,M^	3,211,075,818	26.46/35	88.47 (scaffolds)	149,353,191 (4.65%)	1,452,404,484 (45.23%)	17,758
CHM1_PacBio_r2^L^	2,996,426,293	26.90/30	88.02	205,559,250 (6.86%)	1,541,211,387 (51.43%)	17,657
CHM1_1.1^S,B^	3,037,866,619	50.36/20	—	157,426,845 (5.18%)	1,417,977,130 (46.68%)	18,040
NA12878_single^L,M^	3,176,574,379	26.83/37	88.26	168,652,649 (5.31%)	1,545,168,387 (48.64%)	6,610
NA12878_Allpaths^S^	2,786,258,565	12.08/67	82.89	90,343,965 (3.24%)	1,250,655,296 (44.89%)	16,995
HuRef^C^	2,844,000,504	17.66/48	85.85	134,317,812 (4.72%)	1,411,487,301 (49.63%)	16,968
Mongolian^S^	2,881,945,563	7.63/111	86.54	121,384,034 (4.21%)	1,399,420,366 (48.56%)	17,189
YH_2.0^S^	2,911,235,363	20.52/39	86.31	127,254,909 (4.37%)	1,397,013,571 (47.99%)	17,125
African^S^	2,676,008,911	0.062/11,689	69.47	55,830,170 (2.09%)	968,988,149 (36.21%)	9,167

NGS, next-generation sequencing. Major sequencing and mapping data used in the assembly are marked by superscript letters: C, chain-terminating Sanger sequences; B, indexed BAC end sequences; L, long reads; M, genome maps; S, NGS short reads.

**Table 3:** Summary of SVs in eight human assemblies compared with GRCh38.

**Assembly**	**Total SVs**	**Novel SVs (insertions and deletions only; %)**	**SVs in repetitive regions (%)**	**SVs in segmentally duplicated regions (%)**	**Assembly specific SVs (insertions and deletions only; %)**	**SVs shared with the CHM1 PacBio read mapping results (insertions and deletions only; %)**
KOREF_C^S,L,M^	9,838	8,392 (85.7)	6,992 (71.1)	912 (9.3)	6,691 (68.3)	955 (9.7)
Mongolian^S^	12,830	10,775 (87.7)	8,929 (69.6)	1,242 (9.7)	9,101 (74.1)	834 (6.8)
YH_2.0^S^	5,027	4,664 (93.8)	4,119 (81.9)	633 (12.6)	3,063 (61.6)	148 (3.0)
CHM1_PacBio_r2^L^	3,454	3,130 (92.0)	2,340 (67.7)	1,002 (29.0)	2,448 (72.0)	301 (8.8)
CHM1_1.1^S,B^	3,926	3,258 (83.7)	2,848 (72.5)	394 (10.0)	2,800 (71.9)	487 (12.5)
NA12878_single^L,M^	4,859	4,171 (86.7)	3,339 (68.7)	1,041 (21.4)	3,492 (72.6)	400 (8.3)
NA12878_Allpaths^S^	5,179	4,649 (91.0)	4,014 (77.5)	378 (7.3)	3,787 (74.1)	269 (5.3)
African^S^	10,772	10,026 (94.0)	8,362 (77.6)	425 (3.9)	8,935 (83.8)	212 (2.0)

NGS, next-generation sequencing. Major sequencing and mapping data used in the assembly are marked by superscript letters: B, indexed BAC end sequences; L, long reads; M, genome maps; S, NGS short reads.
