# Corrigendum: An ethnically relevant consensus Korean reference genome is a step towards personal reference genomes

**DOI:** 10.1038/ncomms16168

**Published:** 2017-10-09

**Authors:** Yun Sung Cho, Hyunho Kim, Hak-Min Kim, Sungwoong Jho, JeHoon Jun, Yong Joo Lee, Kyun Shik Chae, Chang Geun Kim, Sangsoo Kim, Anders Eriksson, Jeremy S. Edwards, Semin Lee, Byung Chul Kim, Andrea Manica, Tae-Kwang Oh, George M. Church, Jong Bhak

Nature Communications 7: Article number: 13637 ; DOI: 10.1038/ncomms13637 (2016): Published 11 24 2016; Updated 10 09 2017.

The original version of this Article contained an error in the email address of the corresponding author George Church. The correct email is gchurch@genetics.med.harvard.edu. The error has been corrected in the HTML and PDF versions of the Article.

