# Supplementary material for: An ethnically relevant consensus Korean reference genome is a step towards personal reference genomes
Source: Nat Commun. 2016 Nov 24;7:13637. doi: 10.1038/ncomms13637 (PMC5123046; doi:10.1038/ncomms13637)
Supplement: Supplementary Information — Supplementary Figures 1-15 and Supplementary Table 1-39 [file ncomms13637-s1.pdf]

Supplementary Information

Supplementary Figures

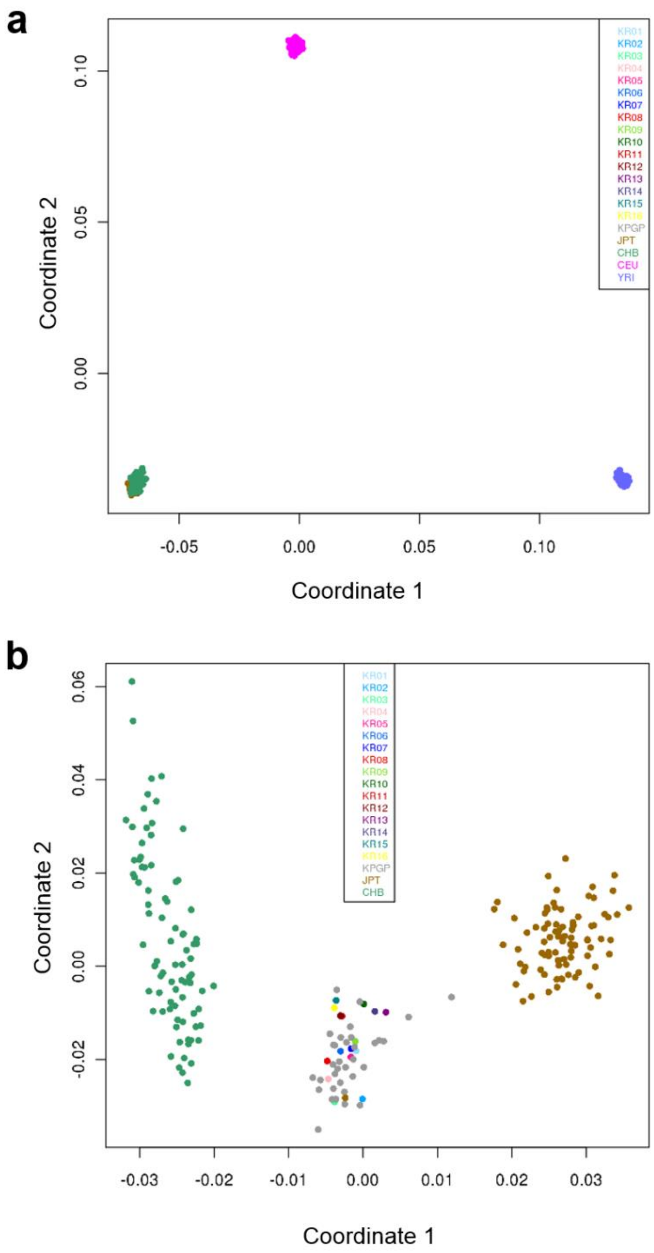

Supplementary Figure 1 | MDS plot of 445 human samples. (**a**) MDS plots of the 16 donors (KR) were drawn by comparing to other 34 Koreans (KPGP), 86 Japanese (JPT), 84 Chinese (CHB), 112 Caucasians (CEU), and 113 Africans (YRI) using 90,462 SNV markers. (**b**) MDS plot among Koreans, Chinese, and Japanese using 72,578 SNV markers. The span of the genetic distance of the 16 did not fall outside the common Korean population range.

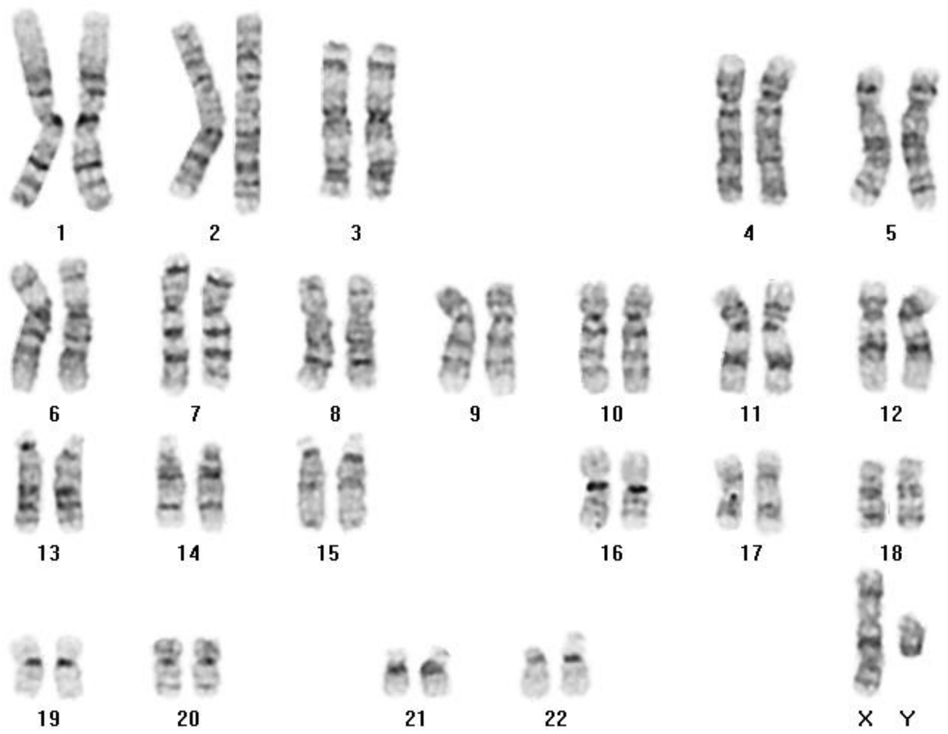

Supplementary Figure 2 | G-banded karyotype of the Korean genome. There were no abnormalities in the chromosomes (2n=46).

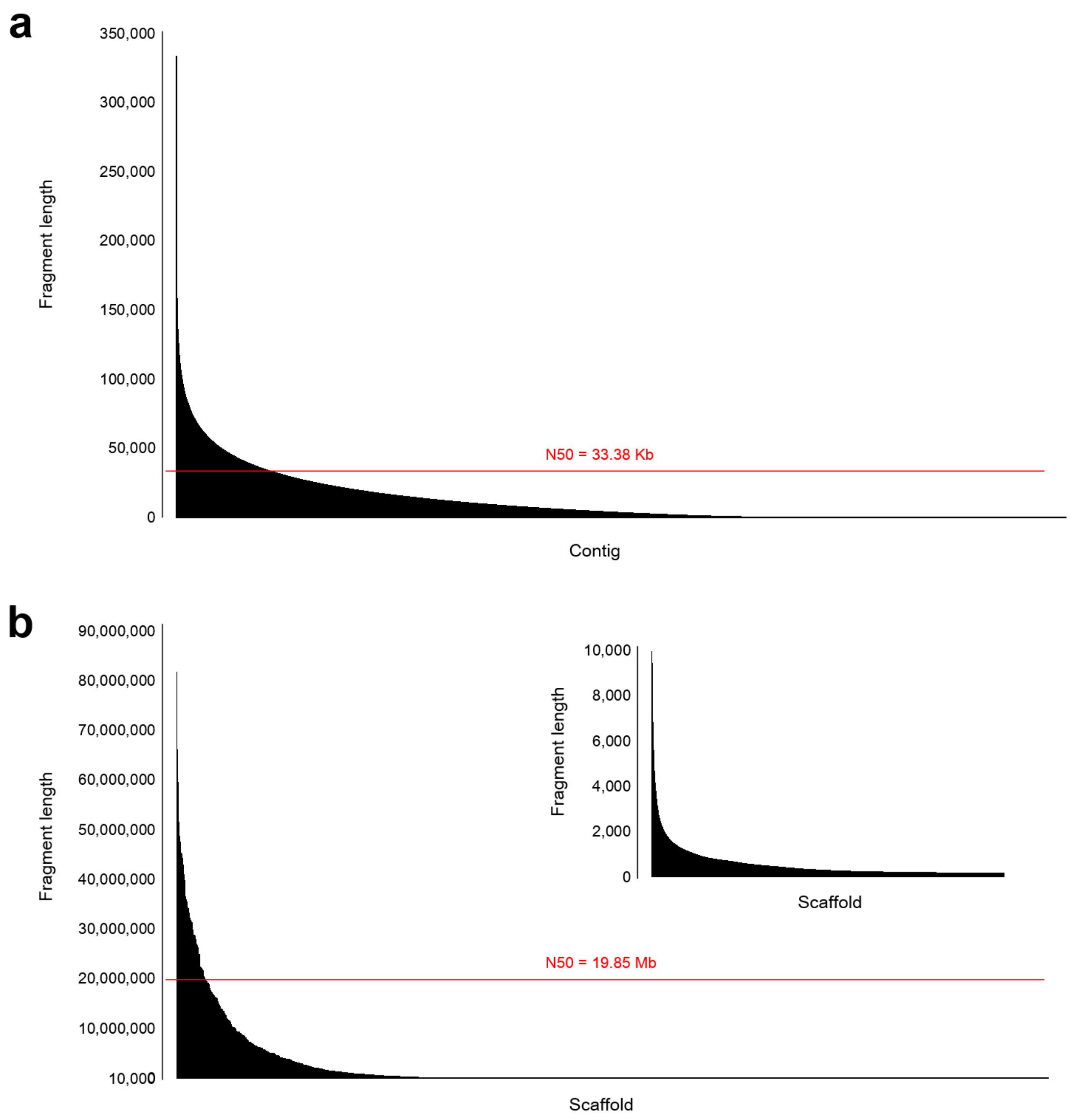

Supplementary Figure 3 | Length distribution of KOREF_S assembled fragments. (**a**) KOREF_S contig using only NGS short read data. (**b**) KOREF_S scaffold using only NGS short read data. Fragments (contigs/scaffolds) were sorted by their lengths.

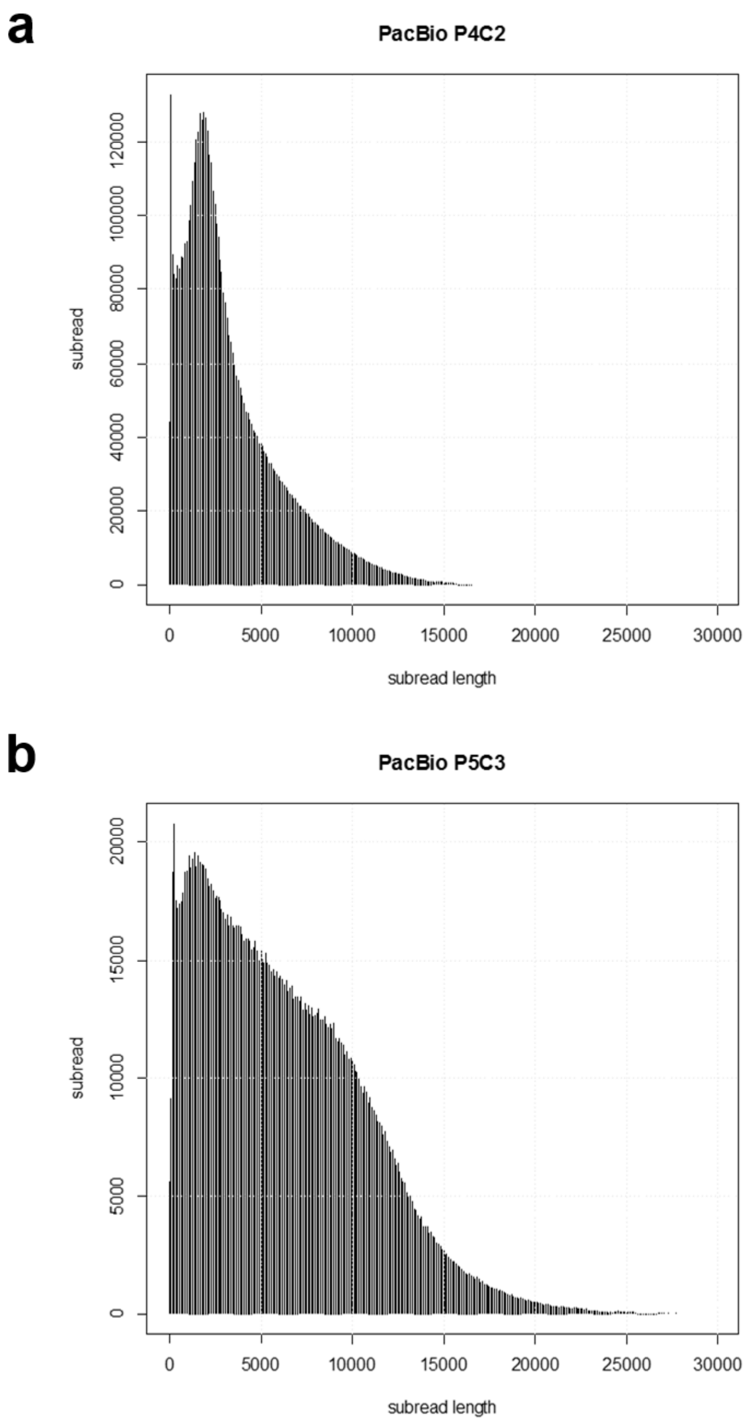

Supplementary Figure 4 | Length distribution of PacBio RSII DNA sequence reads. (**a**) PacBio RSII P4C2. (**b**) PacBio RSII P5C3.

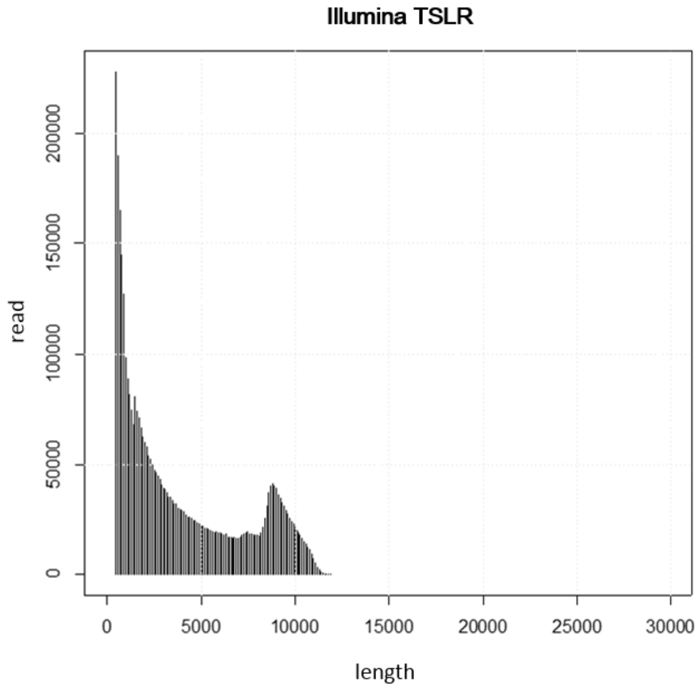

Supplementary Figure 5 | Length distribution of Illumina TruSeq synthetic long reads

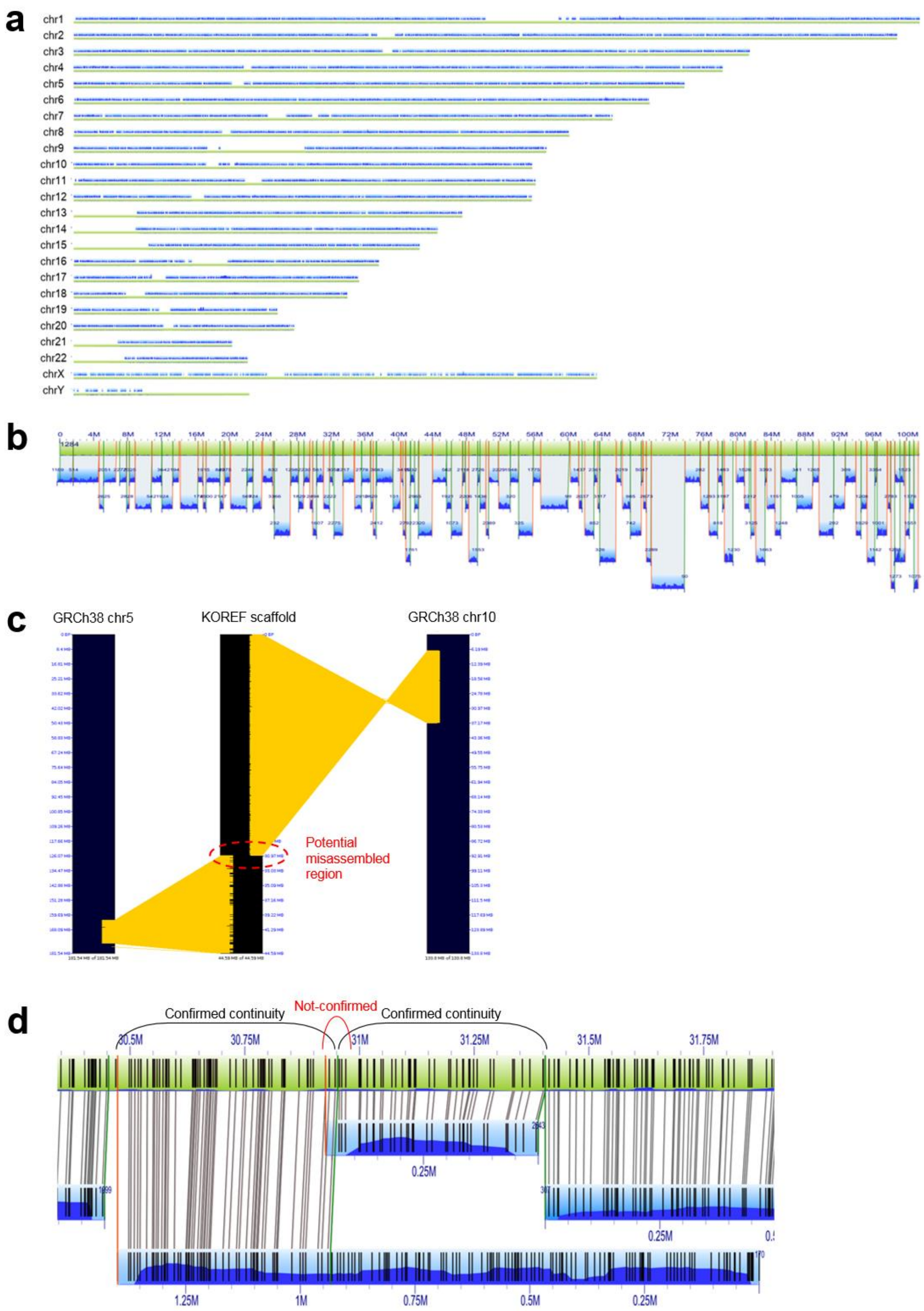

Supplementary Figure 6 | Assessment of scaffold assembly using BioNano genome mapping data. (**a**) Overall view of BioNano consensus maps compared to KOREF_S assembly. Green bars indicate KOREF_S scaffolds, and blues are assembled BioNano genome maps. (**b**) The longest KOREF_S scaffold (~101 Mb) confirmed by BioNano consensus maps. (**c**) An example of potentially misassembled regions. (**d**) Confirmation of the potentially misassembled region in the panel (c) using the consensus maps.

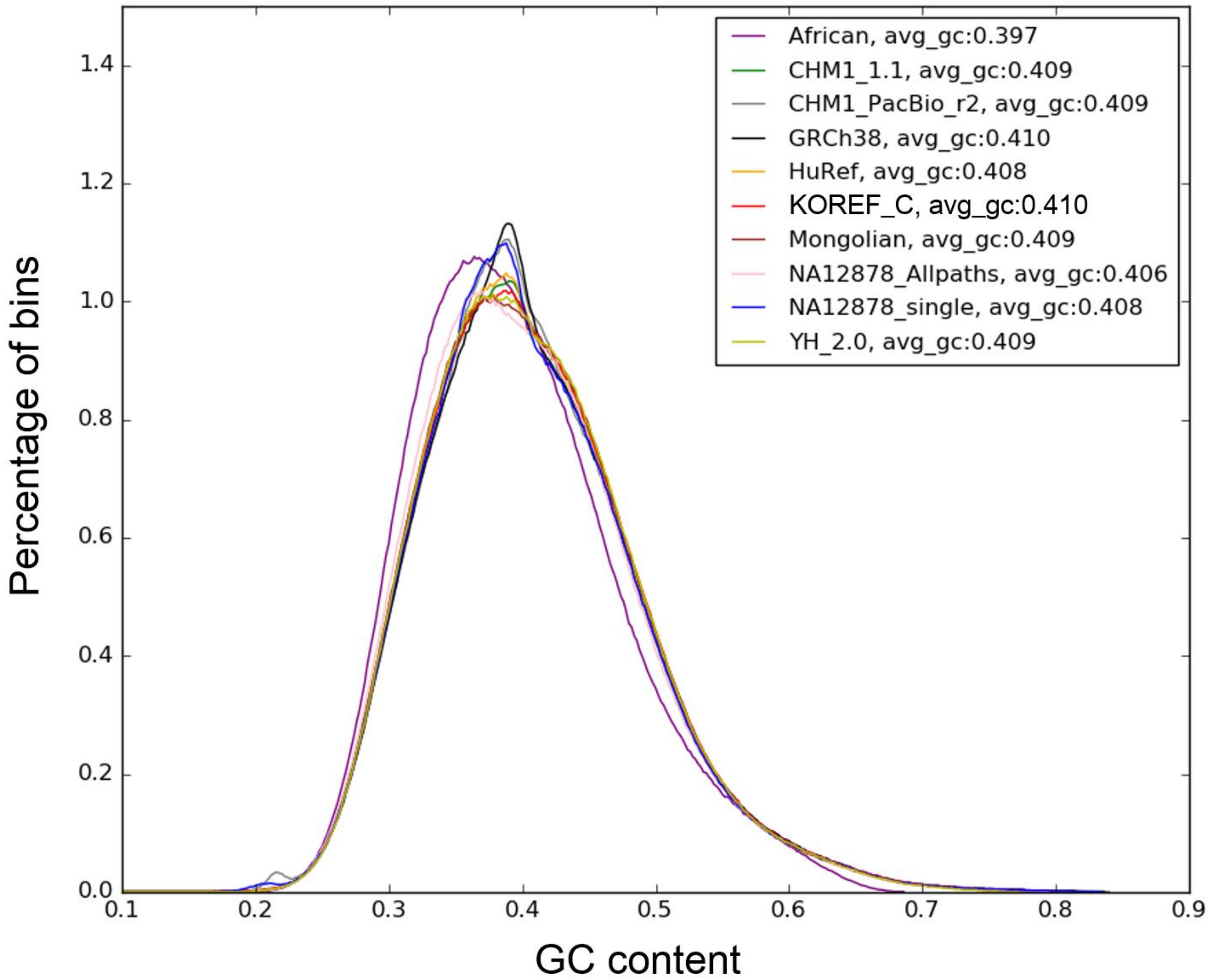

Supplementary Figure 7 | GC content distributions in the human genome assemblies. The *x*-axis is GC content, and the *y*-axis is the proportion of the bin count with the specified GC content.

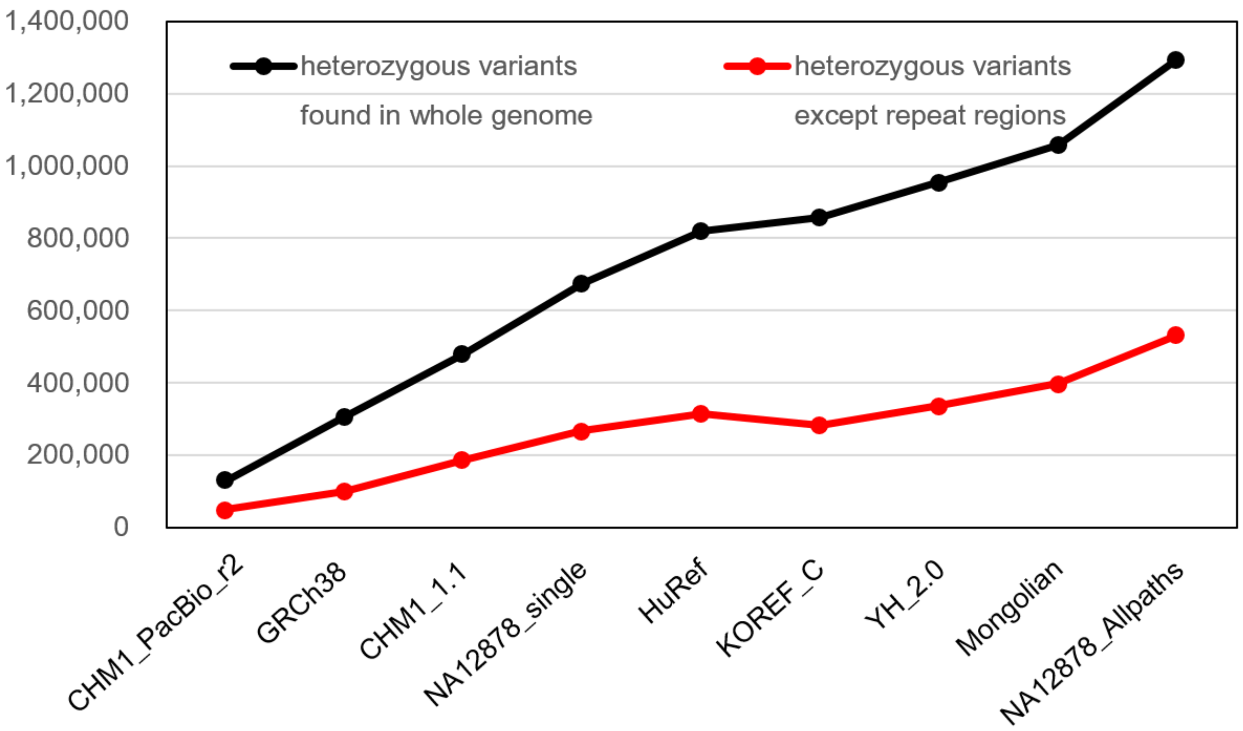

Supplementary Figure 8 | Numbers of heterozygous variants found in re-sequencing data from a single haplotype (CHM1) genome

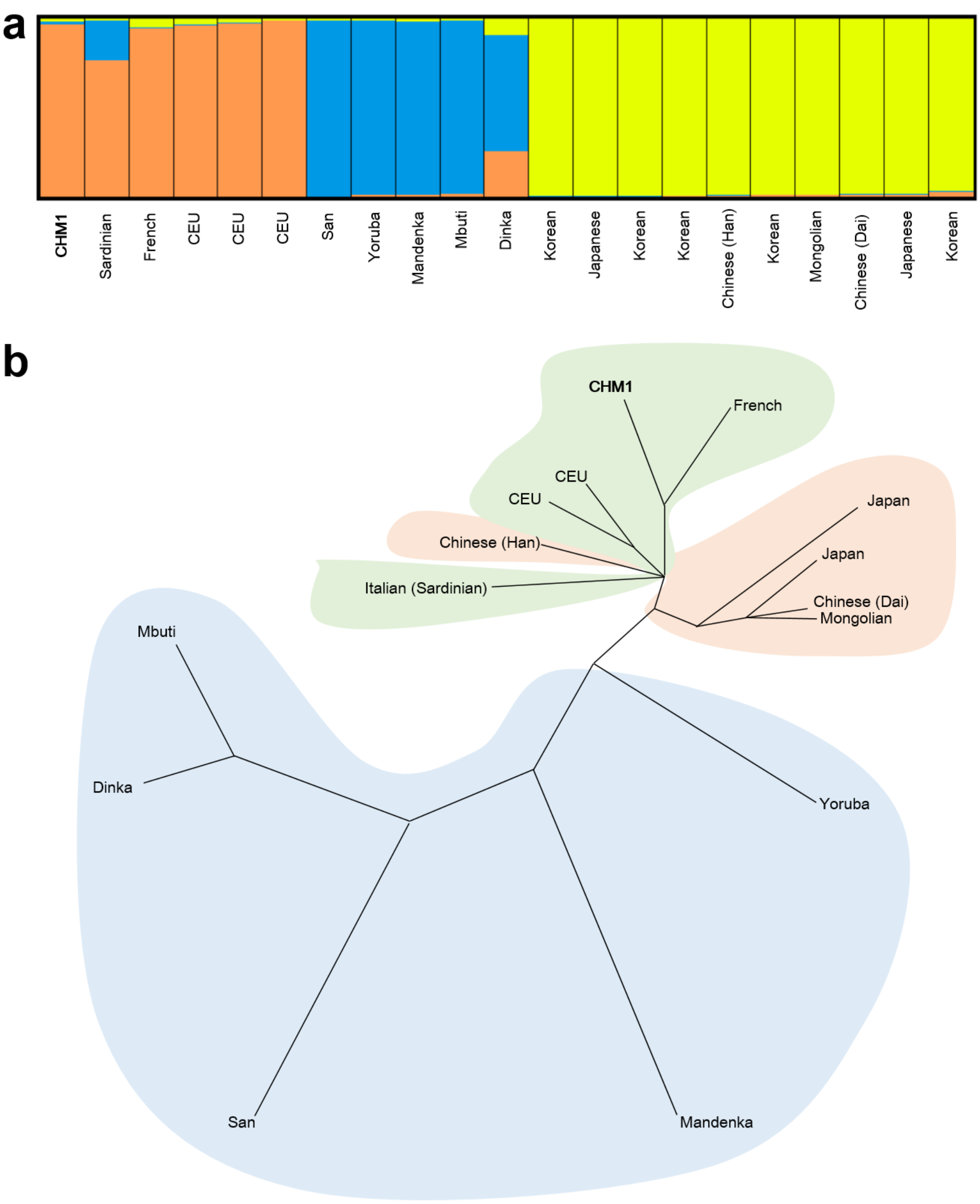

Supplementary Figure 9 | CHM1 ethnicity confirmation. (**a**) STRUCTURE analysis using 47 ancestry-sensitive DNA markers in autosomes. For *K*=3, CHM1 is grouped together with Europeans. (**b**) Mitochondrial DNA (mtDNA) sequence comparison. The mtDNA sequences were generated by mapping CHM1's Illumina short reads into GRCh38 mtDNA sequences and building consensus sequences.

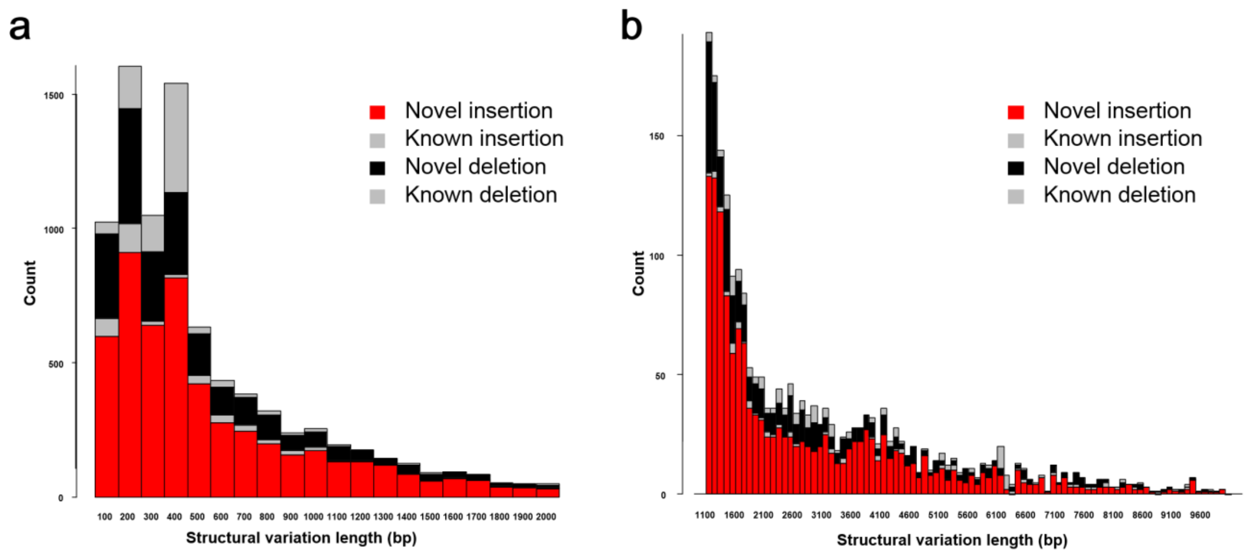

Supplementary Figure 10 | Length distributions of KOREF_C structural variations compared to GRCh38. (**a**) Structural variation lengths range from 50bp to 2 Kb. (**b**) Structural variation lengths range from 1 Kb to 10 Kb.

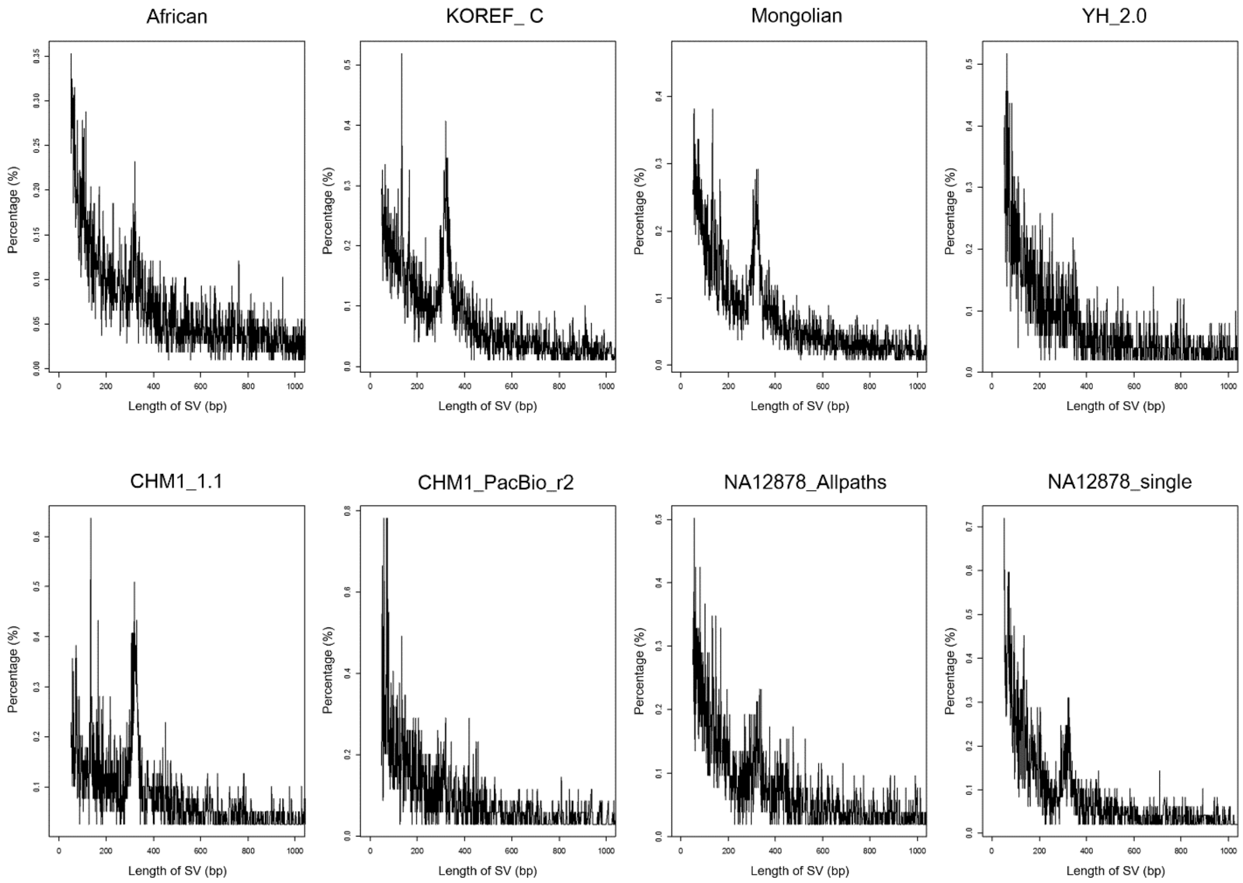

Supplementary Figure 11 | Length distributions of structural variations found in human assemblies compared to GRCh38

a. Structural variations shared by only two assemblies

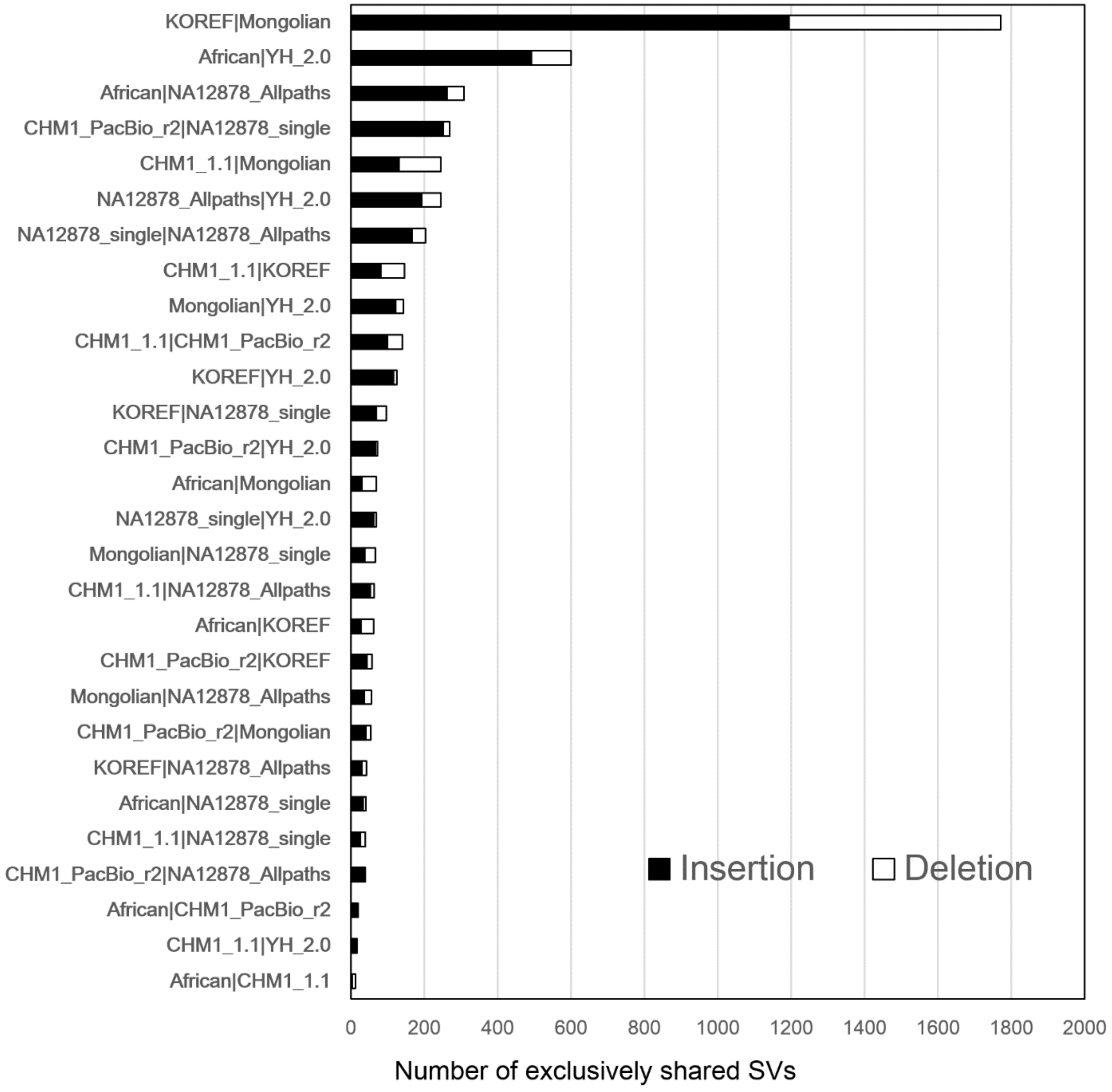

b. Structural variations shared by only three assemblies

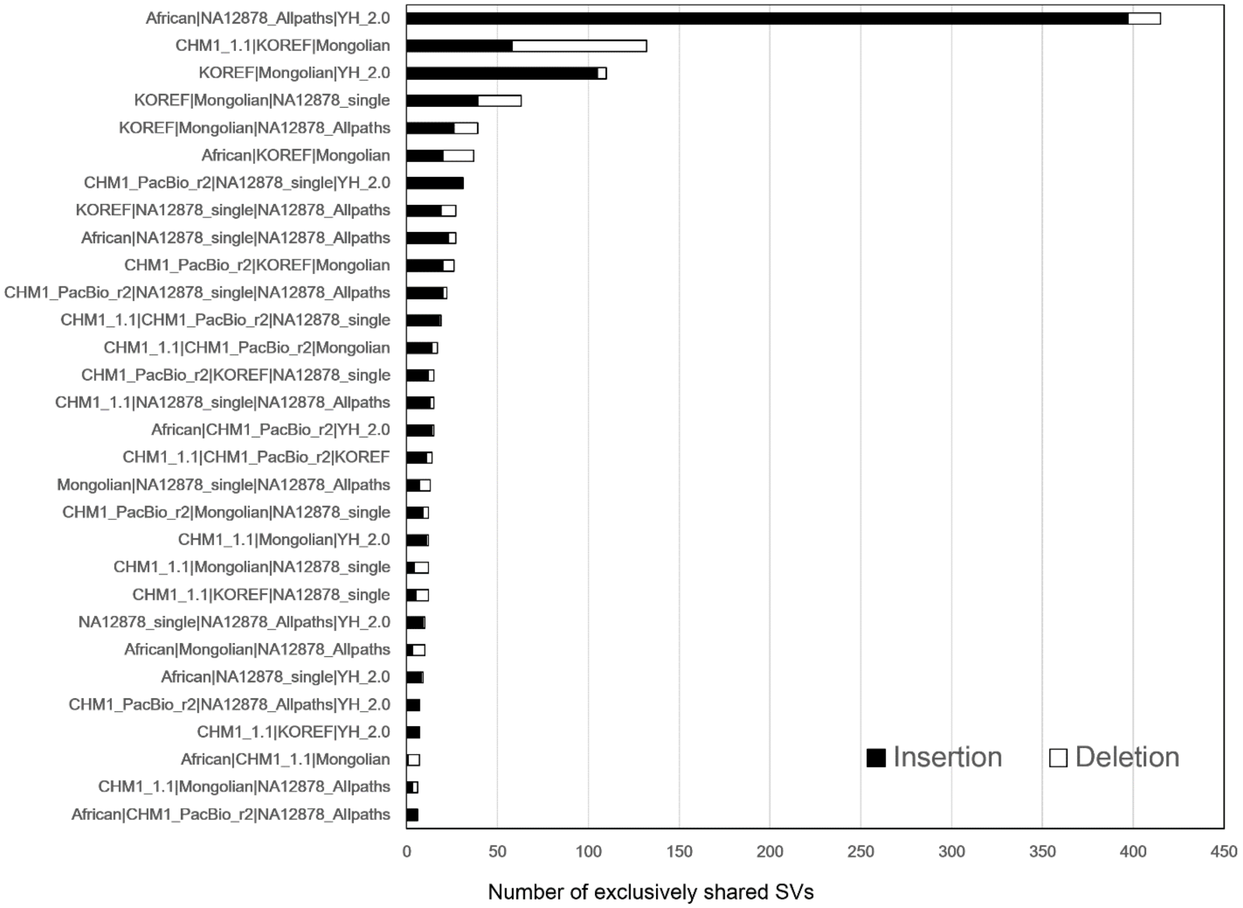

Supplementary Figure 12 | Exclusively shared structural variations among human assembly sets. Structural variations shared (reciprocally 50 % covered) by only denoted assemblies (*y*-axis: assembly sets) were considered in this figure. KOREF indicates KOREF_C. (**a**) Structural variations shared by only two assemblies. (**b**) Structural variations shared by only three assemblies. Only cases with five or more shared structural variations are shown.

a. Exclusively shared insertions excluding repetitive and segmentally-duplicated regions

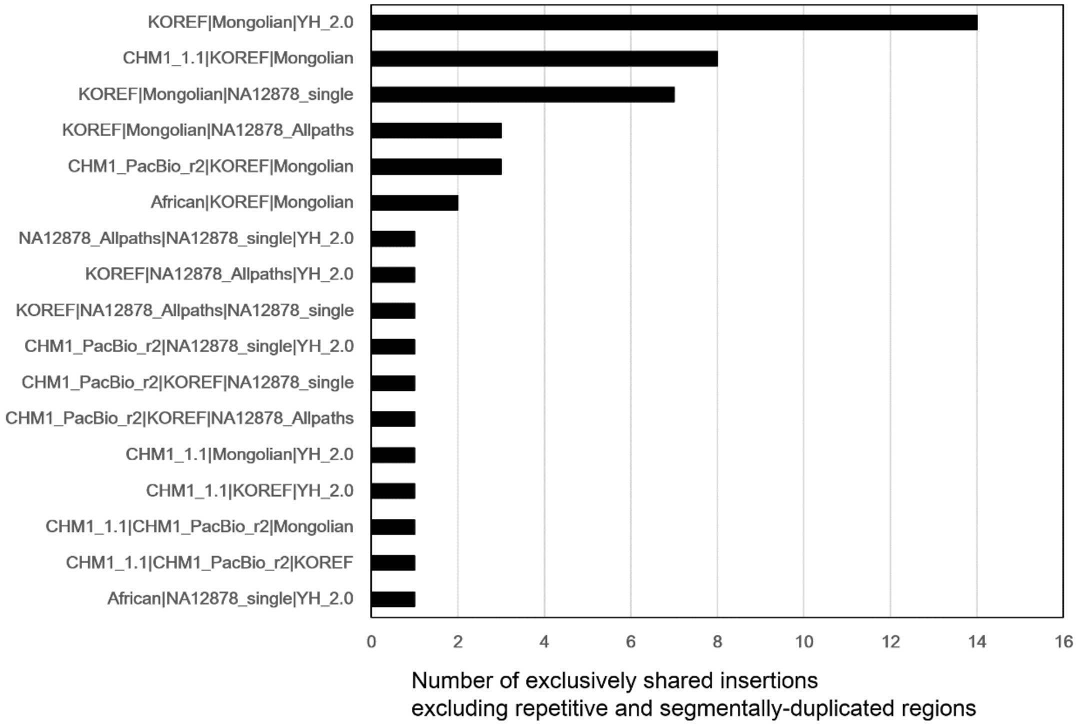

b. Exclusively shared deletions excluding repetitive and segmentally-duplicated regions

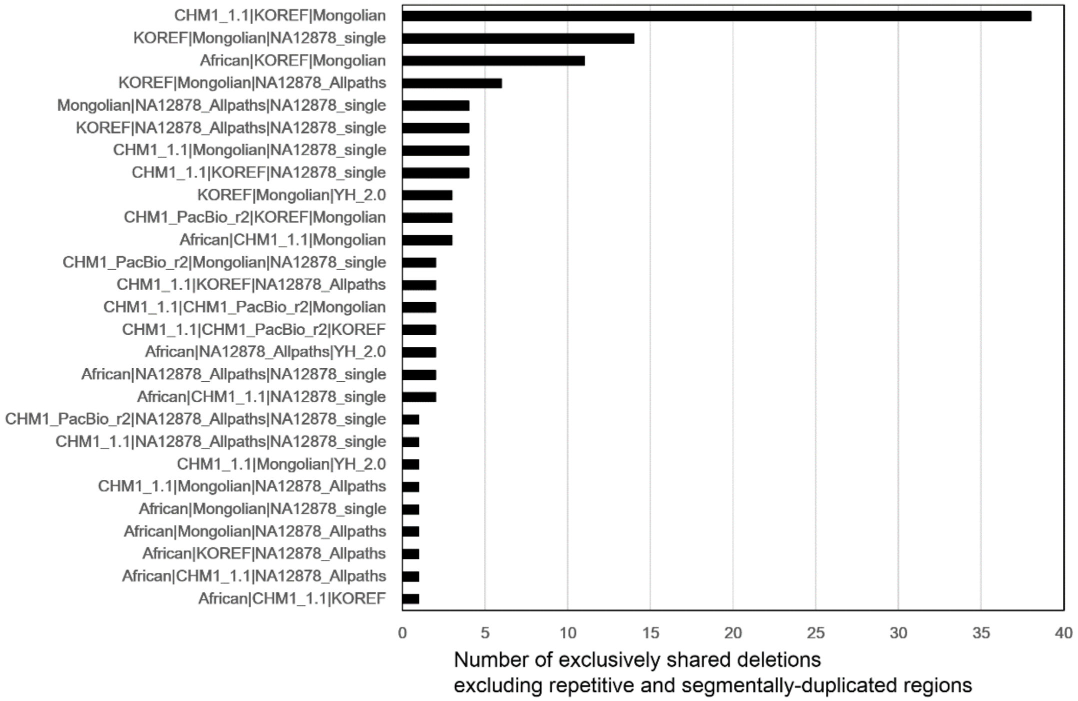

Supplementary Figure 13 | Exclusively shared structural variations excluding repetitive and segmentally-duplicated regions. Structural variations shared by only three assemblies were considered in this figure (reciprocally 50 % covered). KOREF indicates KOREF_C. (**a**) Exclusively shared insertions. (**b**) Exclusively shared deletions.

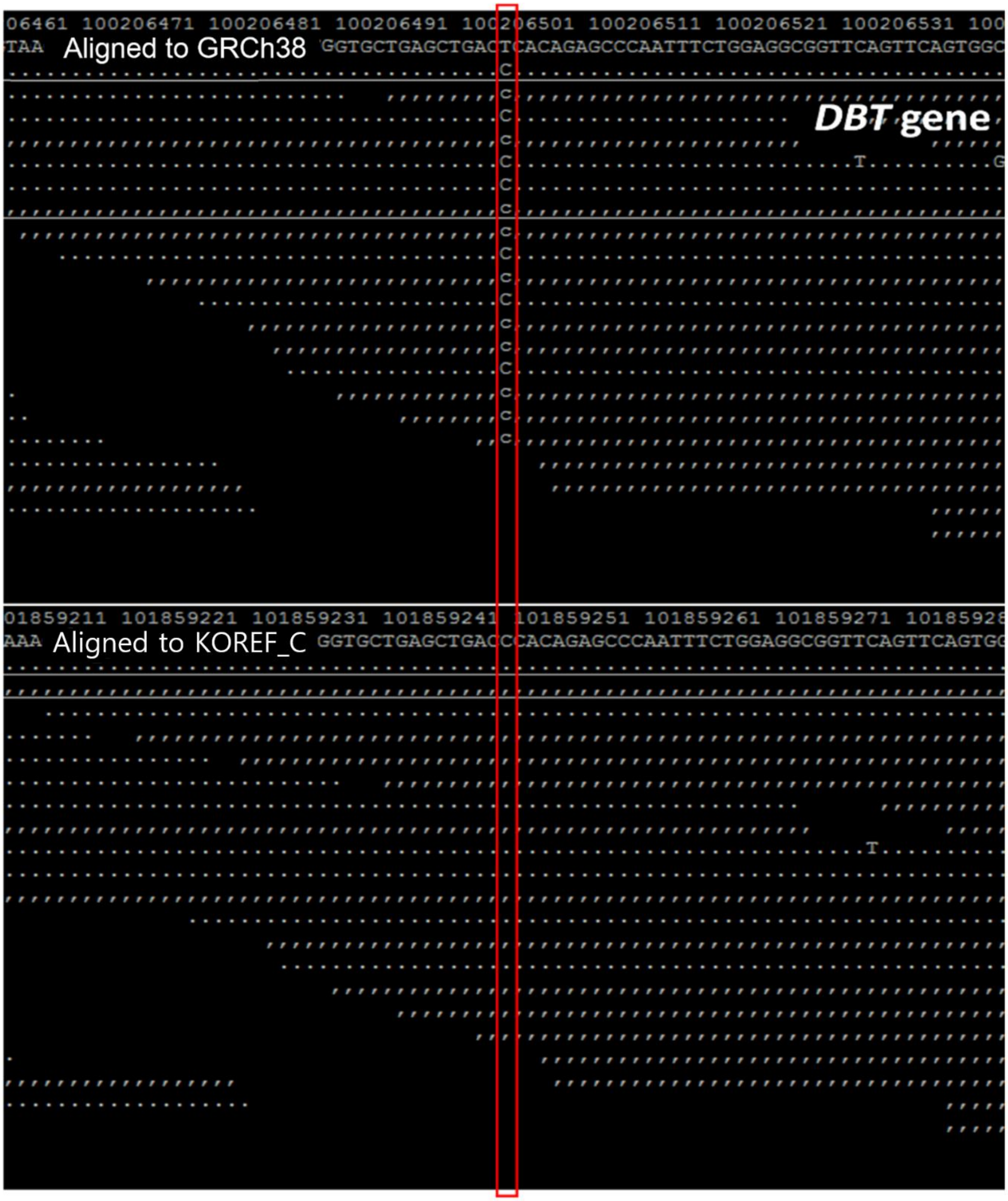

Supplementary Figure 14 | An example of variants that were called against GRCh38, but not KOREF_C. The 13 nsSNVs that are known as disease- and phenotype-associated were verified by visual inspection of short reads alignments.

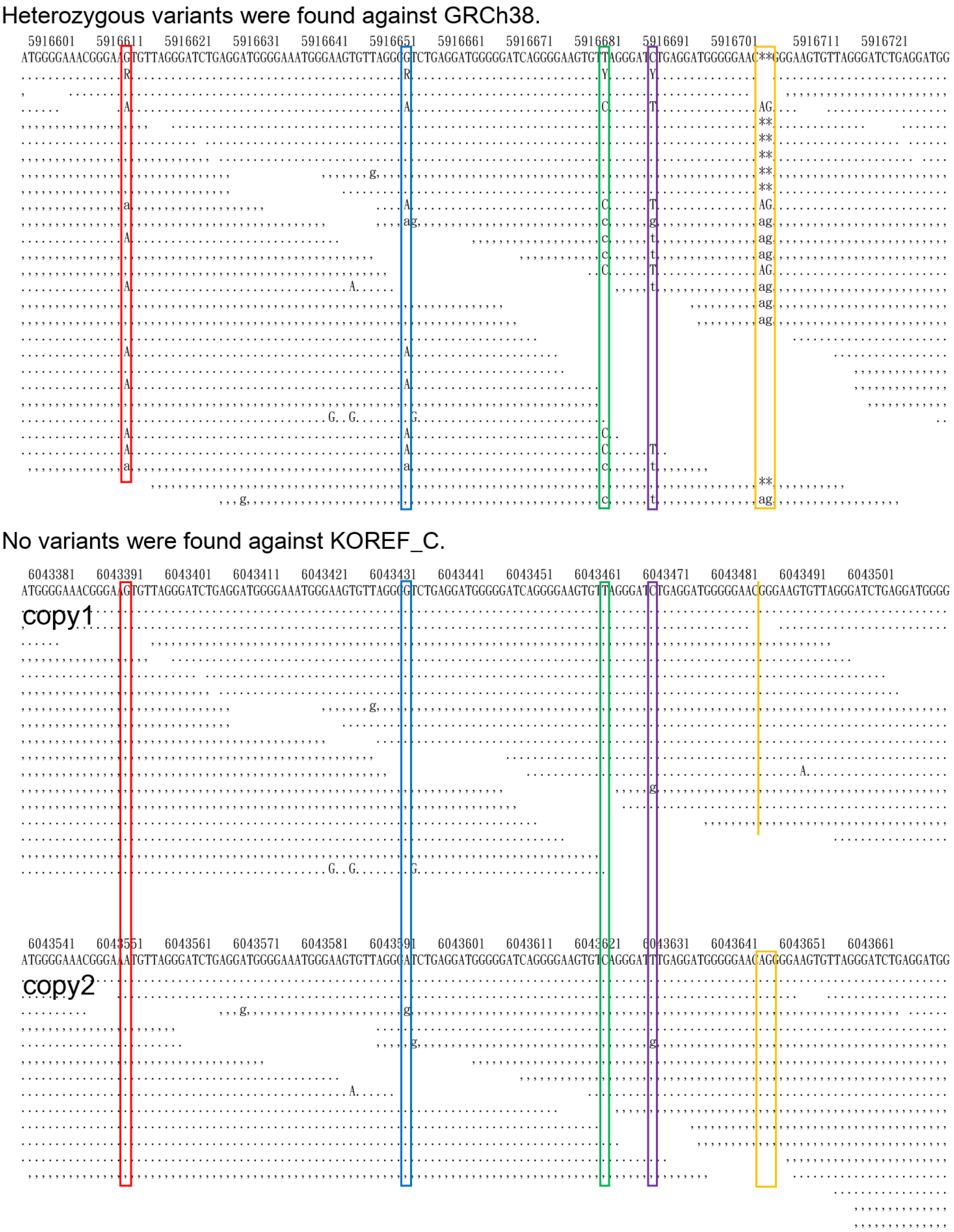

Supplementary Figure 15 | An example of genotype reconstruction difference in GRCh38 and KOREF_C. GRCh38 has one copy region, but KOREF_C has two copies for the same region. Heterozygous variants that may be caused by the copy number difference were not detected when using KOREF_C.

Supplementary Tables

Supplementary Table 1 | Quality control results of the 16 blood sample donors in this study

ID	Conc_ Quant-iT (ng/ul)	Vol. (ul)	Fluorescence amount (ug)	Conc_UV (ng/ul)	260/ 280	260/ 230	UV amount
KR-01	127	45	5.72	195.9	1.78	2.07	8.82
KR-02	137	48	6.58	208.1	1.79	2.19	9.99
KR-03	159	49	7.79	234.0	1.78	2.02	11.47
KR-04	376	43	16.17	467.1	1.81	2.14	20.09
KR-05	200	49	9.80	286.3	1.81	2.18	14.03
KR-06	270	41	11.07	525.7	1.82	2.05	21.55
KR-07	328	40	13.12	579.9	1.82	2.00	23.20
KR-08	131	41	5.37	183.5	1.81	2.17	7.52
KR-09	101	42	4.24	172.5	1.80	2.13	7.25
KR-10	125	43	5.38	192.8	1.80	2.18	8.29
KR-11	103	43	4.43	156.8	1.81	2.12	6.74
KR-12	129	43	5.55	177.8	1.81	2.12	7.65
KR-13	98.9	52	5.14	152.4	1.81	1.82	7.92
KR-14	164	43	7.05	238.7	1.82	2.17	10.26
KR-15	186	43	8.00	275.1	1.80	2.14	11.83
KR-16	147	980	144.06	228.8	1.79	2.10	224.22

Supplementary Table 2 | Statistics regarding Illumina whole-genome shotgun sequence

Type	Insert size	Read length (bp)	Number of read pairs	Total data (Gb)	Sequence depth (×)	
Short-insert size libraries	170bp	101	254,562,947	51.42	16.59	48.69
			246,624,330	49.82	16.07	
			246,007,078	49.70	16.03	
	500bp	101	246,418,836	49.78	16.06	46.71
			230,109,465	46.48	14.99	
			240,361,539	48.55	15.66	
	700bp	101	207,193,678	41.85	13.50	39.17
			188,159,956	38.01	12.26	
			205,873,335	41.59	13.41	
Long-mate pair libraries	2Kb	101	196,290,337	39.65	12.79	38.22
			232,858,099	47.04	15.17	
			157,507,662	31.82	10.26	
	5Kb	101	152,201,289	30.74	9.92	32.81
			177,874,430	35.93	11.59	
			173,383,733	35.02	11.30	
	10Kb	101	205,215,277	41.45	13.37	40.05
			209,859,354	42.39	13.67	
			199,617,521	40.32	13.01	
	15Kb	101	156,336,183	31.58	10.19	30.65
			166,036,249	33.54	10.82	
			147,927,209	29.88	9.64	
	20Kb	101	181,506,276	36.66	11.83	34.72
			177,434,679	35.84	11.56	
			173,929,946	35.13	11.33	
Total			4,773,289,408	964.19	311.02	311.02

Supplementary Table 3 | Statistics regarding filtered and trimmed whole-genome shotgun sequence

Type	Insert size	Read length (bp)	Number of read pairs	Total data (Gb)	Sequence Depth (×)	
Short-insert size libraries	170bp	90	238,901,578	43.00	13.87	40
			225,934,916	40.67	13.12	
			224,145,725	40.35	13.01	
	500bp	90	220,100,704	39.62	12.78	37.57
			207,716,033	37.39	12.06	
			219,165,329	39.45	12.73	
	700bp	90	189,043,000	34.03	10.98	32.24
			173,545,699	31.24	10.08	
			192,535,557	34.66	11.18	
Long-mate pair libraries	2Kb	49	102,368,796	10.03	3.24	9.64
			118,485,351	11.61	3.75	
			83,704,400	8.20	2.65	
	5Kb	49	74,199,538	7.27	2.35	8.08
			93,060,115	9.12	2.94	
			88,156,446	8.64	2.79	
	10Kb	49	52,521,514	5.15	1.66	5.03
			54,759,429	5.37	1.73	
			51,874,811	5.08	1.64	
	15Kb	49	60,904,413	5.97	1.93	5.3
			55,631,632	5.45	1.76	
			51,042,581	5.00	1.61	
	20Kb	49	20,374,949	2.00	0.64	2.08
			26,561,512	2.60	0.84	
			19,032,195	1.87	0.60	
Total			2,843,766,223	433.77	139.94	139.94

Supplementary Table 4 | Statistics regarding 23-mer analysis results

K-mer size	*K*-mer total number	Peak depth	Genome size (bp)	Used base (bp)	Used reads number	Depth coverage (×)	Average read length (bp)	*K*-mer species number
23	87,989,560,976	29	3,034,122,792	116,456,771,880	1,293,964,132	38.3824	90	5,689,732,938

Supplementary Table 5 | *In silico* restriction enzyme selection on the KOREF_S scaffolds. *SpeI* enzyme was used for the KOREF_S whole genome optical map building.

Enzyme	Usable% 5Kb-20Kb	Usable% 6Kb-15Kb	Usable% 6Kb-12Kb	Ave. Frags size (kb)	# of Frags > 100kb	Max Frag size (Kb)
AflII	25.12	10.31	10.07	4.58	4	117.49
BamHI	94.94	82.36	72.76	8.08	19	159.82
KpnI	98.76	91.89	69.64	10.35	50	154.09
NcoI	17.1	3.37	3.35	3.85	0	84.46
NheI	98.08	89.26	65.1	10.67	62	149.61
SpeI	94.8	73.17	67.9	7.44	63	196.12
BglII	7.01	2.12	2.07	3.79	1	104.69
EcoRI	7.86	2.87	2.85	3.65	0	71.37
MluI	0.76	0.23	0.09	130.62	9422	1529.97
NdeI	12.35	6.4	6.21	3.25	3	105.73
PvuII	2.2	0.4	0.4	2.7	3	149.7
XbaI	9.27	3.33	3.26	3.64	3	147.38
XhoI	26.46	11.1	4.88	23.64	2612	372.38

Supplementary Table 6 | OpGen single molecule restriction map statistics

Summary of SMRM data	Maps used in analysis
Total Size (Gb)	745.51
Number of Molecules	2,071,951
Average Size of Molecules (Kb)	359.81
Minimum molecule size (Kb)	250
Average Size of Fragments (Kb)	13.24

Supplementary Table 7 | Scaffold joining results using optical mapping data

Scaffold1	size1(kb)	strand1	Scaffold2	size2 (kb)	strand2	Gap (kb)	Score
SCAFFOLD317	1022.416	1	SCAFFOLD743	842.84	-1	14.466	99.4236
SCAFFOLD210	11746.639	1	SCAFFOLD940	551.059	-1	12.506	97.8962
SCAFFOLD244	882.071	1	SCAFFOLD150	8643.747	1	-4.539	92.9372
SCAFFOLD532	495.294	1	SCAFFOLD280	1697.743	-1	16.755	87.2892
SCAFFOLD103	8759.181	1	SCAFFOLD431	2527.758	1	4.325	80.7857
SCAFFOLD8	18209.097	1	SCAFFOLD122	5972.151	1	17.543	69.7056
SCAFFOLD79	778.308	1	SCAFFOLD292	913.969	1	1.636	66.4837
SCAFFOLD77	4752.716	1	SCAFFOLD89	4287.167	1	0.067	64.7672
SCAFFOLD89	4287.167	1	SCAFFOLD140	10524.263	1	-5.698	62.3363
SCAFFOLD63	8355.854	-1	SCAFFOLD163	6250.598	1	14.348	55.3254
SCAFFOLD356	1363.545	-1	SCAFFOLD743	842.84	1	71.197	55.2638
SCAFFOLD70	19845.87	1	SCAFFOLD42	6341.468	1	202.32	54.2056
SCAFFOLD110	6289.28	1	SCAFFOLD170	3210.067	1	2.994	53.1726
SCAFFOLD19	29018.184	1	SCAFFOLD364	2266.538	1	39.026	47.5055
SCAFFOLD485	689.059	1	SCAFFOLD343	2303.617	1	57.217	43.8511
SCAFFOLD428	511.544	1	SCAFFOLD31	2851.399	-1	116.431	43.2197
SCAFFOLD126	5708.801	1	SCAFFOLD219	1429.49	-1	85.562	43.2175
SCAFFOLD353	2639.995	1	SCAFFOLD15	2258.516	1	10.722	38.5231
SCAFFOLD91	5409.31	1	SCAFFOLD63	8355.854	-1	190.878	38.2565
SCAFFOLD169	5101.962	1	SCAFFOLD653	227.433	1	12.551	32.557
SCAFFOLD87	12817.817	-1	SCAFFOLD212	3045.171	1	16.396	29.8232
SCAFFOLD264	14081.586	1	SCAFFOLD575	626.29	1	25.872	28.7976
SCAFFOLD24	15566.053	1	SCAFFOLD3	13712.728	1	-0.342	28.4213
SCAFFOLD502	381.379	-1	SCAFFOLD533	1080.224	1	0.859	27.1306
SCAFFOLD1072	619.532	1	SCAFFOLD189	12056.91	-1	51.438	26.8774
SCAFFOLD246	13977.981	-1	SCAFFOLD206	20601.118	1	5.588	24.7277
SCAFFOLD322	4940.238	1	SCAFFOLD201	6752.265	1	2.859	23.4562
SCAFFOLD337	286.159	1	SCAFFOLD787	520.497	1	25.873	22.9017
SCAFFOLD103	8759.181	-1	SCAFFOLD11	5130.215	1	0.002	22.6392
SCAFFOLD85	5575.593	1	SCAFFOLD302	1599.441	-1	-5.59	21.6902
SCAFFOLD82	5897.044	1	SCAFFOLD43	28037.362	1	-0.311	21.4608
SCAFFOLD533	1080.224	1	SCAFFOLD27	4154.534	-1	5.276	21.2813
SCAFFOLD246	13977.981	1	SCAFFOLD112	34485.537	-1	-3.432	19.0796
SCAFFOLD392	875.318	1	SCAFFOLD289	1425.336	-1	6.962	18.2247
SCAFFOLD142	7148.482	1	SCAFFOLD59	5549.968	1	-0.24	18.0723
SCAFFOLD7	40570.24	-1	SCAFFOLD199	16436.955	1	10.033	17.6323
SCAFFOLD233	3346.963	1	SCAFFOLD147	30048.452	-1	3.123	17.3518
SCAFFOLD377	1560.501	1	SCAFFOLD233	3346.963	1	7.023	16.3624
SCAFFOLD455	3872.703	1	SCAFFOLD85	5575.593	1	-3.332	16.165
SCAFFOLD872	333.932	1	SCAFFOLD243	2305.143	1	82.932	16.098
SCAFFOLD350	999.02	-1	SCAFFOLD142	7148.482	1	236.727	16.0549
SCAFFOLD197	9499.216	1	SCAFFOLD12	2823.635	1	-6.936	15.8702
SCAFFOLD569	387.885	-1	SCAFFOLD119	1305.15	1	5.536	15.3893
SCAFFOLD434	1008.885	1	SCAFFOLD423	472.166	-1	16.713	15.3473
SCAFFOLD153	18967.221	1	SCAFFOLD353	2639.995	1	29.897	14.2316
SCAFFOLD161	943.876	1	SCAFFOLD87	12817.817	-1	147.087	14.2259
SCAFFOLD98	48842.997	1	SCAFFOLD235	10164.153	1	6.502	13.9087
SCAFFOLD232	242.678	1	SCAFFOLD218	444.904	1	0.834	13.8088
SCAFFOLD296	792.382	1	SCAFFOLD35	1500.96	1	37.211	13.7568
SCAFFOLD54	14806.717	1	SCAFFOLD214	5135	1	4.606	13.3133
SCAFFOLD502	381.379	1	SCAFFOLD222	4068.33	-1	11.1	12.7174
SCAFFOLD100	6592.548	1	SCAFFOLD359	2048.679	-1	27.867	12.3654
SCAFFOLD49	36078.134	-1	SCAFFOLD100	6592.548	1	0.002	12.3407
SCAFFOLD243	2305.143	1	SCAFFOLD940	551.059	1	8.69	12.3289
SCAFFOLD146	6416.747	1	SCAFFOLD40	20409.372	1	4.306	11.054
SCAFFOLD350	999.02	1	SCAFFOLD570	524.193	-1	51.022	10.734
SCAFFOLD39	8825.901	-1	SCAFFOLD104	7398.895	1	-11.052	10.6812
SCAFFOLD306	1232.982	1	SCAFFOLD99	3038.256	-1	-6.299	10.29
SCAFFOLD42	6341.468	1	SCAFFOLD263	2671.43	1	52.694	10.0097
SCAFFOLD638	678.726	1	SCAFFOLD79	778.308	1	116.653	9.9301
SCAFFOLD86	16308.764	1	SCAFFOLD16	19543.299	-1	-1.287	9.8459
SCAFFOLD170	3210.067	1	SCAFFOLD306	1232.982	1	254.75	9.683
SCAFFOLD120	19315.79	1	SCAFFOLD38	81906.269	1	5.027	9.6118
SCAFFOLD649	661.586	1	SCAFFOLD570	524.193	1	576.918	9.3685
SCAFFOLD392	875.318	-1	SCAFFOLD169	5101.962	1	19.408	9.1531
SCAFFOLD178	423.463	1	SCAFFOLD28	12121.666	-1	67.343	9.12
SCAFFOLD364	2266.538	1	SCAFFOLD74	3948.894	1	4.863	9.0136

Supplementary Table 8 | PacBio RSII long reads statistics

a. PacBio P4C2

Size	Number of bases (bp)	Number of reads	Mean length (bp)
~2kb	2,200,375,125	2,023,326	1,088
~3kb	2,598,138,881	1,054,927	2,463
~4kb	2,253,729,183	650,819	3,463
~5kb	1,993,913,569	445,503	4,476
~6kb	1,868,335,867	341,037	5,478
~7kb	1,692,679,373	261,244	6,479
~8kb	1,490,151,540	199,293	7,477
~9kb	1,264,147,938	149,166	8,475
~10kb	1,025,254,470	108,261	9,470
10kb~	2,404,653,532	202,921	11,850
Total	18,791,379,478	5,436,497	3,457

b. PacBio P5C3

Region	Number of bases (bp)	Number of reads	Mean length (bp)
~2kb	376,691,922	352,650	1,068
~3kb	448,189,058	179,744	2,493
~4kb	581,090,138	166,158	3,497
~5kb	707,030,086	157,272	4,496
~6kb	815,006,427	148,315	5,495
~7kb	905,881,157	139,481	6,495
~8kb	978,965,060	130,607	7,496
~9kb	1,063,290,046	125,158	8,496
~10kb	1,084,089,752	114,232	9,490
10kb~	5,347,185,274	406,019	13,170
Total	12,307,418,920	1,919,636	6,411

Supplementary Table 9 | Illumina TruSeq synthetic long reads statistics

Region	Number of bases (bp)	Number of reads	Mean length (bp)
~2kb	1,745,885,089	1,627,362	1,073
~3kb	1,227,839,348	498,112	2,465
~4kb	1,200,052,670	345,449	3,474
~5kb	1,170,624,980	261,313	4,480
~6kb	1,141,935,546	208,259	5,483
~7kb	1,132,652,780	174,578	6,488
~8kb	1,358,992,691	181,044	7,506
~9kb	2,532,232,743	294,819	8,589
~10kb	2,879,791,577	304,656	9,453
10kb~	1,910,098,184	181,128	10,546
Total	16,300,105,608	4,076,720	3,998

Supplementary Table 10 | The number of sequence reads for scaffolding generated in this study

	Mate-pairs (read depth: ~20×)	PacBio reads (read depth: ~10×)	TSLRs (read depth: ~5.3×)
The number of read information that can be used for scaffolding	952,677,682	7,356,133	4,076,720
The ratio to mate-pair number	100 %	0.77 %	0.43 %

Supplementary Table 11 | BioNano genome mapping data statistics

	BioNano single molecules	BioNano consensus maps
Total data	210 Gb	-
Single molecule N50	273 Kb	-
Molecules above 150Kb	145 Gb	-
Coverage depth	45 ×	-
Assembly size	-	2.78 Gb
Consensus map N50	-	1.12 Mb

Supplementary Table 12 | Mapping and variants statistics of 40 Korean whole genomes aligned to KOREF_S

Sample ID	Total number of raw reads	Mapped read depth (except 'N')	Read mapping rate (%)	Homozygous SNVs	Homozygous INDELs	Heterozygous SNVs	Heterozygous INDELs	All variants
KPGP-00002	98,317,515,960	27.64	99.29	962,066	146,462	2,958,707	292,082	4,359,317
KPGP-00006	93,448,081,980	24.73	99.28	1,431,527	204,234	2,915,971	276,219	4,827,951
KPGP-00032	112,190,946,660	30.36	99.29	1,444,163	215,475	2,955,815	296,145	4,911,598
KPGP-00033	108,196,466,760	29.95	99.30	1,406,058	211,651	2,961,708	297,035	4,876,452
KPGP-00039	101,141,448,400	30.19	99.16	1,391,102	212,028	2,991,047	315,678	4,909,855
KPGP-00056	111,361,334,200	32.24	99.34	1,419,373	230,317	3,100,438	340,429	5,090,557
KPGP-00086	102,626,322,600	29.88	99.34	1,423,097	228,216	3,074,640	335,156	5,061,109
KPGP-00125	118,670,365,980	33.12	99.31	1,438,747	211,687	2,932,733	291,074	4,874,241
KPGP-00127	118,883,354,760	32.81	99.33	1,416,527	206,959	2,948,523	288,104	4,860,113
KPGP-00128	117,849,278,700	32.76	99.29	1,407,530	208,532	2,941,634	292,805	4,850,501
KPGP-00129	107,124,150,780	29.96	99.28	1,440,746	203,979	2,908,731	271,108	4,824,564
KPGP-00131	120,142,829,340	33.36	99.29	1,432,319	211,261	2,970,372	289,604	4,903,556
KPGP-00132	122,237,363,160	33.93	99.30	1,411,276	210,946	2,946,694	297,988	4,866,904
KPGP-00134	119,540,641,320	32.54	99.28	1,416,157	207,904	2,931,855	288,305	4,844,221
KPGP-00136	114,984,689,940	30.71	99.30	1,429,777	204,804	2,940,492	274,170	4,849,243
KPGP-00137	118,027,255,140	32.97	99.28	1,403,331	207,581	2,940,643	289,256	4,840,811
KPGP-00138	123,868,546,380	33.39	99.32	1,398,902	207,327	2,938,964	289,045	4,834,238
KPGP-00139	105,730,760,700	29.32	99.28	1,397,287	207,216	2,918,240	291,707	4,814,450
KPGP-00141	111,508,577,820	31.41	99.24	1,405,400	207,892	2,926,108	288,957	4,828,357
KPGP-00142	125,024,326,200	32.62	99.29	1,443,241	211,075	2,943,175	292,818	4,890,309
KPGP-00144	127,001,127,600	33.96	99.30	1,422,369	211,512	2,973,541	296,396	4,903,818
KPGP-00145	111,861,808,380	31.18	99.29	1,438,003	210,730	2,953,375	293,052	4,895,160
KPGP-00205-B01-G	123,835,438,866	37.24	98.41	1,422,423	221,835	3,072,207	332,313	5,048,778
KPGP-00220	106,317,727,560	28.21	99.28	1,411,132	201,485	2,931,702	284,397	4,828,716
KPGP-00227	115,164,844,920	34.39	99.30	1,419,518	217,159	3,039,274	308,248	4,984,199
KPGP-00228	112,898,405,520	33.34	99.30	1,455,818	221,343	3,052,488	303,008	5,032,657
KPGP-00230	110,458,697,940	32.86	99.31	1,414,415	214,448	3,031,789	301,182	4,961,834
KPGP-00232	109,620,112,860	32.01	99.29	1,442,223	214,897	3,020,544	292,548	4,970,212
KPGP-00233	107,091,428,940	32.08	99.27	1,421,451	216,917	3,014,334	302,473	4,955,175
KPGP-00235	114,400,539,900	34.74	99.31	1,414,391	218,911	3,047,216	309,518	4,990,036
KPGP-00245-B01-G-PE500	102,078,086,860	31.40	99.11	1,465,527	223,235	3,031,190	322,301	5,042,253
KPGP-00254	122,277,928,000	34.56	99.24	1,427,301	221,720	3,080,569	313,709	5,043,299
KPGP-00255	102,221,657,600	29.67	99.34	1,414,140	227,857	3,083,228	336,527	5,061,752
KPGP-00256	127,033,362,000	36.61	99.35	1,422,753	235,874	3,174,628	355,538	5,188,793
KPGP-00265-B01-G-P500	90,922,729,400	27.53	99.29	1,414,977	216,811	2,964,359	306,126	4,902,273
KPGP-00266-B01-G-P500	91,666,078,800	27.38	99.32	1,374,215	212,665	2,962,424	307,516	4,856,820
KPGP-00269-B01-G-PE500	100,240,975,874	30.81	99.32	1,449,250	219,822	3,052,622	324,886	5,046,580
KPGP-00317-B01-G-PE500	103,075,371,660	26.76	87.15	1,400,454	208,300	3,002,602	306,055	4,917,411
KPGP-00318-B01-G-PE500	101,805,865,370	28.22	95.42	1,440,304	218,383	2,971,844	319,451	4,949,982
KPGP-00319-B01-G-PE500	100,957,938,100	27.77	97.17	1,403,626	213,564	3,063,114	315,785	4,996,089

Supplementary Table 13 | Variations found in KOREF_S mtDNA compared to GRCh38 mtDNA

Position	Ref	Alt	Gene	Variant type	Amino acid Change	dbSNP143
73	A	G	*TRNF*	Upstream variant	-	rs3087742
263	A	G	*TRNF*	Upstream variant	-	rs2853515
310	T	CTC	*TRNF*	Upstream variant	-	rs66492218
489	T	C	*TRNF*	Upstream variant	-	rs28625645
750	A	G	*RNR1*	Noncoding variant	-	rs2853518
1438	A	G	*RNR1*	Noncoding variant	-	rs2001030
2706	A	G	*RNR2*	Noncoding variant	-	rs2854128
3010	G	A	*RNR2*	Noncoding variant	-	rs3928306
3107	N	-	*RNR2*	Noncoding variant	-	-
4769	A	G	*ND2*	Synonymous variant	-	rs3021086
4883	C	T	*ND2*	Synonymous variant	-	rs200763872
5178	C	A	*ND2*	Missense variant	Met237Leu	rs28357984
7028	C	T	*COX1*	Synonymous variant	-	rs2015062
8414	C	T	*ATP8*	Missense variant	Leu17Phe	rs28358884
8701	A	G	*ATP6*	Missense variant	Thr58Ala	rs2000975
8860	A	G	*ATP6*	Missense variant	Thr112Ala	rs2001031
9010	G	A	*ATP6*	Missense variant	Ala162Thr	-
9540	T	C	*COX3*	Synonymous variant	-	rs2248727
10398	A	G	*ND3*	Missense variant	Thr114Ala	rs2853826
10400	C	T	*ND3*	Synonymous variant	-	rs28358278
10873	T	C	*ND4*	Synonymous variant	-	rs2857284
11215	C	T	*ND4*	Synonymous variant	-	rs386419997
11719	G	A	*ND4*	Synonymous variant	-	-
12705	C	T	*ND5*	Synonymous variant	-	-
14668	C	T	*ND6*	Synonymous variant	-	rs28357678
14766	C	T	*CYTB*	Missense variant	Thr7Ile	rs527236041
14783	T	C	*CYTB*	Synonymous variant	-	rs527236042
15043	G	A	*CYTB*	Synonymous variant	-	rs527236043
15148	G	A	*CYTB*	Synonymous variant	-	rs527236206
15184	T	C	*CYTB*	Synonymous variant	-	-
15301	G	A	*CYTB*	Synonymous variant	-	rs527236045
15326	A	G	*CYTB*	Missense variant	Thr194Ala	rs2853508
16223	C	T	*CYTB*	Downstream variant	-	rs2853513
16362	T	C	*CYTB*	Downstream variant	-	rs62581341

Supplementary Table 14 | KOREF_C repeat annotation

	Repbase TEs		*De novo*		Combined	
	Length (bp)	% in Genome	Length (bp)	% in Genome	Length (bp)	% in Genome
DNA	106,469,686	3 %	24,415,664	1 %	108,618,651	3 %
LINE	610,159,517	19 %	536,712,478	17 %	745,903,228	23 %
SINE	390,299,729	12 %	254,443,404	8 %	425,991,881	13 %
LTR	267,766,723	8 %	112,840,399	4 %	270,236,817	8 %
Unknown	837,329	0 %	17,216,396	1 %	18,050,168	1 %
Total	1,450,469,642	45 %	994,936,953	31 %	1,513,511,651	47 %

Supplementary Table 15 | KOREF_C protein-coding gene prediction

Gene set	Gene number	Average transcript length (bp)	Average CDS length (bp)	Average exon per gene	Average exon length (bp)	Average intron length (bp)
Homology (Human)	18,564	51,797.23	1,701.15	9.80	173.59	5,847.84
de novo	18,988	51,291.92	1,485.38	9.16	162.07	6,099.12
mtDNA	13	876.54	876.54	1.00	876.54	-
Combined	20,400	49,584.30	1,635.35	9.41	173.76	5,847.28

Supplementary Table 16 | KOREF_C-specific novel sequence identification

a. KOREF_S short reads mapped to each human genome assembly

	Mapped KOREF_S short reads	Unmapped KOREF_S short reads	Mapped reads (out of unmapped reads to other human assemblies) to KOREF_C assembly	Total length of novel sequences (bp; the regions with length ≥100bp and covered by at least three reads)
GRCh38	-	4,087,416	1,340,733	4,676,384
Microbial sequences	2,485,935	1,601,481		
CHM1_1.1	261,251	1,340,230	1,080,569	4,012,692
HuRef	900,070	440,160	182,060	1,305,352
African	74,658	365,502	108,958	1,024,687
Mongolian	40,008	325,494	69,725	890,472
YH	3,705	321,789	67,385	875,820

b. Length distribution of KOREF_C novel sequences

Length	Number of fragments (≥100bp)
100 – 500bp	2,531
501 – 1,000bp	240
1,001 – 5,000bp	89
5,001bp – 10 Kb	1
Above 10 Kb	1
Total length of novel sequences (bp)	875,820

Supplementary Table 17 | Global assembly statistics of human assemblies. Major sequencing and mapping data used in the assembly are marked by superscript letters: NGS short reads, S; long reads, L; genome maps, M; indexed BAC end sequences, B; chain-terminating Sanger sequences; C.

Statistics	GRCh38^{C}	KOREF_C^{S,L,M}	CHM1_ PacBio_r2^{L}	CHM1_1.1^{S,B}	NA12878_ single^{L,M}
Assembly level	Chromosome	Chromosome	Contig	Chromosome	Scaffold
Total sequence length	3,209,286,105	3,211,075,818	2,996,426,293	3,037,866,619	3,176,574,379
Total assembly gap length	159,970,007	297,934,127	0	210,229,812	146,352,286
Gaps between scaffolds	349	4,495	-	225	0
Number of scaffolds	735	4,481	-	163	18,903
Scaffold N50	67,794,873	26,457,717	-	50,362,920	26,834,081
Scaffold L50	16	35	-	20	37
Number of contigs	1,385	198,871	3,641	40,828	21,235
Contig N50	56,413,054	47,858	26,899,841	143,936	1,557,716
Contig L50	19	17,749	30	5,635	532
Total number of chromosomes and plasmids	25	25	0	23	0

Statistics	NA12878_ Allpaths^{S}	HuRef^{C}	Mongolian^{S}	YH_2.0^{S}	African^{S}
Assembly level	Scaffold	Chromosome	Scaffold	Scaffold	Scaffold
Total sequence length	2,786,258,565	2,844,000,504	2,881,945,563	2,911,235,363	2,676,008,911
Total assembly gap length	171,353,127	34,429,377	58,452,127	105,204,230	592,227,090
Gaps between scaffolds	0	1,396	0	0	0
Number of scaffolds	11,393	4,530	221,013	125,643	314,786
Scaffold N50	12,084,118	17,664,250	7,632,466	20,520,932	62,478
Scaffold L50	67	48	111	39	11,689
Number of contigs	231,194	71,333	321,009	361,157	5,313,377
Contig N50	23,924	108,431	56,244	20,516	887
Contig L50	30,971	7,164	14,915	40,005	642,142
Total number of chromosomes and plasmids	0	24	0	0	0

Supplementary Table 18 | GRCh38 genome recovery rates of human assemblies. Whole genome alignment approach was used to calculate GRCh38 genome recovery rates of human assemblies. Major sequencing and mapping data used in the assembly are marked by superscript letters: NGS short reads, S; long reads, L; genome maps, M; chain-terminating Sanger sequences; C.

Assembly	GRCh38 length (bp)	Assembly length (bp)	Total alignment results (including duplicated alignments)		Non-redundant alignment results (excluding duplicated alignments)	
			Length of aligned regions (bp)	GRCh38 coverage (%)	Length of aligned regions (bp)	GRCh38 coverage (%)
KOREF_S_scaffold[S,L,M]	3,209,286,105	2,944,499,428	2,956,077,148	92.11	2,839,274,905	88.47
KOREF_S_contig[S,L,M]	3,209,286,105	2,913,213,215	2,944,669,829	91.75	2,755,264,778	85.85
CHM1_PacBio_r2[L]	3,209,286,105	2,996,426,293	2,968,736,981	92.50	2,824,727,975	88.02
NA12878_single[L,M]	3,209,286,105	3,176,574,379	2,948,546,881	91.88	2,832,488,088	88.26
NA12878_Allpaths[S]	3,209,286,105	2,786,258,565	2,753,492,425	85.80	2,660,094,223	82.89
HuRef_contig[C]	3,209,286,105	2,809,571,127	2,942,411,659	91.68	2,755,302,479	85.85
Mongolian[S]	3,209,286,105	2,881,945,563	2,916,062,756	90.86	2,777,307,567	86.54
YH_2.0[S]	3,209,286,105	2,911,235,363	2,885,254,871	89.90	2,769,798,873	86.31
African[S]	3,209,286,105	2,676,008,911	2,354,016,286	73.35	2,229,410,403	69.47

Supplementary Table 19 | Predicted segmentally-duplicated and repetitive sequence regions in human assemblies. Homology search was used to identify segmentally-duplicated and repetitive regions. Major sequencing and mapping data used in the assembly are marked by superscript letters: NGS short reads, S; long reads, L; genome maps, M; indexed BAC end sequences, B; chain-terminating Sanger sequences; C.

Assembly	Assembly length	SD length	SD %	Repeat length	Repeat %
GRCh38[C]	3,209,286,105	212,777,868	6.63	1,564,209,365	48.74
CHM1_PacBio_r2[L]	2,996,426,293	205,559,250	6.86	1,541,211,387	51.43
NA12878_single[L,M]	3,176,574,379	168,652,649	5.31	1,545,168,387	48.64
CHM1_1.1[S,B]	3,037,866,619	157,426,845	5.18	1,417,977,130	46.68
KOREF_C[S,L,M]	3,211,075,818	149,353,191	4.65	1,452,404,484	45.23
KOREF_S_scaffold[S]	2,921,901,481	139,246,009	4.77	1,438,015,194	49.22
HuRef[C]	2,844,000,504	134,317,812	4.72	1,411,487,301	49.63
YH_2.0[S]	2,911,235,363	127,254,909	4.37	1,397,013,571	47.99
Mongolian[S]	2,881,945,563	121,384,034	4.21	1,399,420,366	48.56
NA12878_Allpaths[S]	2,786,258,565	90,343,965	3.24	1,250,655,296	44.89
African[S]	2,676,008,911	55,830,170	2.09	968,988,149	36.21

Supplementary Table 20 | Predicted protein-coding genes in human assemblies. Homology search was used to identify RefSeq protein-coding genes. Major sequencing and mapping data used in the assembly are marked by superscript letters: NGS short reads, S; long reads, L; genome maps, M; indexed BAC end sequences, B; chain-terminating Sanger sequences; C.

# of genes in RefSeq	# of intact genes in RefSeq (without genes having premature stop codons)	Assembly	# of searched genes by TblastN (E-value > 1E-05, Best hit only)	# of gene models by Exonerate prediction (at least 50% of the maximal score obtainable for query)	# of detected RefSeq genes (by removing genes having premature stop codons)
20,196	20,135	African^{S}	19,924	12,282	9,167
		CHM1_1.1^{S,B}	20,167	19,848	18,040
		CHM1_PacBio_r2^{L}	20,176	19,888	17,657
		HuRef^{C}	20,165	19,578	16,968
		KOREF_C^{S,L,M}	20,181	19,748	17,758
		KOREF_S_scaffold^{S}	20,179	19,719	17,750
		Mongolian^{S}	20,174	19,458	17,189
		NA12878_Allpaths^{S}	20,117	18,978	16,995
		NA12878_single^{L,M}	20,119	19,482	6,610
		YH_2.0^{S}	20,161	19,241	17,125

Supplementary Table 21 | Structural variations found in human assemblies compared to GRCh38. Major sequencing and mapping data used in the assembly are marked by superscript letters: NGS short reads, S; long reads, L; genome maps, M; indexed BAC end sequences, B.

Assembly	Types	Insertion	Deletion	Inversion	Total
KOREF_C^{S,L,M}	No. of Confident SVs	6,397	3,399	42	9,838
	Minimum (bp)	51	51	45	-
	Maximum (bp)	37,813	36,793	44,546	-
Mongolian^{S}	No. of Confident SVs	6,904	5,386	540	12,830
	Minimum (bp)	51	51	90	-
	Maximum (bp)	44,580	44,577	22,225	-
YH_2.0^{S}	No. of Confident SVs	3,896	1,077	54	5,027
	Minimum (bp)	51	51	53	-
	Maximum (bp)	37,683	43,540	39,965	-
CHM1_PacBio_r2^{L}	No. of Confident SVs	2,969	433	52	3,454
	Minimum (bp)	51	51	14	-
	Maximum (bp)	37,524	24,278	50,943	-
CHM1_1.1^{S,B}	No. of Confident SVs	2,415	1,477	34	3,926
	Minimum (bp)	51	51	44	-
	Maximum (bp)	35,612	18,511	16,592	-
NA12878_single^{L,M}	No. of Confident SVs	3,896	914	49	4,859
	Minimum (bp)	51	51	23	-
	Maximum (bp)	43,701	16,093	20,342	-
NA12878_Allpaths^{S}	No. of Confident SVs	4,012	1,097	70	5,179
	Minimum (bp)	51	51	53	-
	Maximum (bp)	40,018	7,860	46,762	-
African^{S}	No. of Confident SVs	7,991	2,673	108	10,772
	Minimum (bp)	51	51	12	-
	Maximum (bp)	24,657	23,065	39,807	-

Supplementary Table 22 | Structural variations found in genic regions. Major sequencing and mapping data used in the assembly are marked by superscript letters: NGS short reads, S; long reads, L; genome maps, M; indexed BAC end sequences, B.

Region	KOREF_C^{S,L,M}	Mongolian^{S}	YH_ 2.0^{S}	CHM1_ PacBio_r2^{L}	CHM1_1.1^{S,B}	NA12878_ single^{L,M}	NA12878_ Allpaths^{S}	African^{S}
CDS	403	559	122	134	173	149	192	288
UTR	193	277	60	48	92	70	105	115
Intron	2,958	3,388	783	884	1,444	1,184	1,261	1,629
Gene (Total)	2,985	3,427	792	899	1,466	1,205	1,281	1,650

Supplementary Table 23 | Structural variations in repetitive regions. Major sequencing and mapping data used in the assembly are marked by superscript letters: NGS short reads, S; long reads, L; genome maps, M; indexed BAC end sequences, B.

Assembly	Total SVs	SVs in repeats	SVs in non-repeats	The percentage of SVs in repeats
KOREF_C^{S,L,M}	9,838	6,992	2,846	71.1
Mongolian^{S}	12,830	8,929	3,901	69.6
YH_2.0^{S}	5,027	4,119	908	81.9
CHM1_PacBio_r2^{L}	3,454	2,340	1,114	67.7
CHM1_1.1^{S,B}	3,926	2,848	1,078	72.5
NA12878_single^{L,M}	4,859	3,339	1,520	68.7
NA12878_Allpaths^{S}	5,179	4,014	1,165	77.5
African^{S}	10,772	8,362	2,410	77.6

Supplementary Table 24 | Structural variations in segmentally-duplicated regions. Major sequencing and mapping data used in the assembly are marked by superscript letters: NGS short reads, S; long reads, L; genome maps, M; indexed BAC end sequences, B.

Assembly	Total SVs	SVs in segmental duplicated regions	SVs not in segmental duplicated regions	The percentage of SVs in segmental duplicated regions
KOREF_C^{S,L,M}	9,838	912	8,926	9.3
Mongolian^{S}	12,830	1,242	11,588	9.7
YH_2.0^{S}	5,027	633	4,394	12.6
CHM1_PacBio_r2^{L}	3,454	1,002	2,452	29.0
CHM1_1.1^{S,B}	3,926	394	3,532	10.0
NA12878_single^{L}	4,859	1,041	3,818	21.4
NA12878_Allpaths^{S}	5,179	378	4,801	7.3
African^{S}	10,772	425	10,347	3.9

Supplementary Table 25 | Novel structural variations found in the human assemblies. Major sequencing and mapping data used in the assembly are marked by superscript letters: NGS short reads, S; long reads, L; genome maps, M; indexed BAC end sequences, B.

	Insertion				Deletion			
	# of insertions	Novel insertions	Known insertions	% of novel insertions	# of deletions	Novel deletions	Known deletions	% of novel deletions
CHM1 PacBio read mapping approach	10,978	10,029	949	91.4	7,071	3,164	3,907	44.7
KOREF_C[S,L,M]	6,397	5,999	398	93.8	3,399	2,393	1,006	70.4
Mongolian[S]	6,904	6,500	404	94.1	5,386	4,275	1,111	79.4
YH_2.0[S]	3,896	3,806	90	97.7	1,077	858	219	79.7
CHM1_PacBio_r2[L]	2,969	2,802	167	94.4	433	328	105	75.8
CHM1_1.1[S,B]	2,415	2,374	41	98.3	1,477	884	593	59.8
NA12878_single[L,M]	3,896	3,633	263	93.2	914	538	376	58.9
NA12878_Allpaths[S]	4,012	3,897	115	97.1	1,097	752	345	68.6
African[S]	7,991	7,893	98	98.8	2,673	2,133	540	79.8

Supplementary Table 26 | Structural variations shared with CHM1 PacBio read mapping results. Major sequencing and mapping data used in the assembly are marked by superscript letters: NGS short reads, S; long reads, L; genome maps, M; indexed BAC end sequences, B.

Assembly	Total SVs (only insertions or deletions)	The number of shared SVs with the CHM1 PacBio read mapping results			
		Shared SVs	Shared Insertions	Shared Deletions	% of shared SVs
KOREF_C[S,L,M]	9,796	955	477	478	9.75
Mongolian[S]	12,290	834	362	472	6.79
YH_2.0[S]	4,973	148	113	35	2.98
CHM1_PacBio_r2[L]	3,402	301	258	43	8.85
CHM1_1.1[S,B]	3,892	487	87	400	12.51
NA12878_single[L,M]	4,810	400	224	176	8.32
NA12878_Allpaths[S]	5,109	269	137	132	5.27
African[S]	10,664	212	50	162	1.99

Supplementary Table 27 | Assembly-specific structural variations. Major sequencing and mapping data used in the assembly are marked by superscript letters: NGS short reads, S; long reads, L; genome maps, M; indexed BAC end sequences, B.

Assembly	Total SVs (only insertions or deletions)	The number of assembly specific SVs	The number of shared SVs with other assemblies	The percentage of the specific SVs
KOREF_C[S,L,M]	9,796	6,691	3,105	68.3
Mongolian[S]	12,290	9,101	3,189	74.1
YH_2.0[S]	4,973	3,063	1,910	61.6
CHM1_PacBio_r2[L]	3,402	2,448	954	72.0
CHM1_1.1[S,B]	3,892	2,800	1,092	71.9
NA12878_single[L,M]	4,810	3,492	1,318	72.6
NA12878_Allpaths[S]	5,109	3,787	1,322	74.1
African[S]	10,664	8,935	1,729	83.8

Supplementary Table 28 | Structural variations that were frequently found only in Asian genomes

chr	SV type	GRCh38 start	GRCh38 end	Asian_ support	Asia_ not_support	Non-Asian_ support	Non-Asian_ not_support	*P*-value	Ensembl Gene	Confirmed by short/ long read alignments
chr20	Insertion	60764249	60764435	11	0	3	7	0.0010	-	Confirm
chr1	Insertion	75619372	75619500	5	6	0	10	0.023	-	Undefinable
chr1	Insertion	1565637	1565733	7	4	1	9	0.017	*SSU72*	Confirm
chr5	Insertion	96535023	96535129	5	6	0	10	0.023	*CAST*	Confirm
chr9	Insertion	86053597	86053801	9	2	3	7	0.024	*GOLM1*	Confirm
chr9	Insertion	4345943	4346583	10	1	3	7	0.0067	-	Confirm
chr6	Insertion	161000000	161000000	6	5	1	9	0.043	-	Confirm
chr11	Insertion	134000000	134000000	10	1	4	6	0.021	-	Confirm
chr4	Insertion	86343248	86343734	7	4	1	9	0.017	*MAPK10*	Confirm
chr12	Insertion	10915394	10916410	7	4	1	9	0.017	*PRH1 , PRH1-PRR4, PRR4*	Confirm
chr6	Insertion	169000000	169000000	7	4	0	10	0.0028	-	Confirm
chr6	Insertion	157000000	157000000	10	1	4	6	0.021	-	Confirm
chr11	Insertion	70951060	70951170	9	2	1	9	0.0016	*SHANK2*	Confirm
chr20	Insertion	35564449	35564597	11	0	3	7	0.0010	-	Confirm
chr6	Insertion	40655117	40655181	11	0	5	5	0.012	-	Confirm
chr7	Deletion	117000000	117000000	11	0	2	8	0.00022	-	Confirm
chr6	Deletion	161000000	161000000	8	3	1	9	0.0058	-	Confirm
chr5	Deletion	9411654	9411968	11	0	4	6	0.0039	*SEMA5A*	Confirm

Supplementary Table 29 | Mapping statistics of 20 individuals from different populations

Sample ID	Nation /tribe	Ethnicity	GRCh38		GRCh38_C		KOREF_S		KOREF_C	
			Mapped read depth (except 'N')	Read mapping rate (%)	Mapped read depth (except 'N')	Read mapping rate (%)	Mapped read depth (except 'N')	Read mapping rate (%)	Mapped read depth (except 'N')	Read mapping rate (%)
HGDP01286	Mandenka	African	35.39	98.64	35.39	98.55	36.78	98.78	36.70	98.78
HGDP00936	Yoruba	African	37.93	98.71	37.93	98.60	39.49	98.86	39.40	98.86
HGDP01036	San	African	37.10	98.82	37.11	98.74	38.55	98.93	38.46	98.93
HGDP00982	Mbuti	African	35.63	98.45	35.65	98.36	35.71	98.56	37.00	98.56
DNK07	Dinka	African	33.66	85.50	33.66	84.25	35.01	85.86	34.94	85.86
HGDP01076	Sardinia	Caucasian	36.51	98.56	36.51	98.45	37.85	98.72	37.78	98.72
HGDP00533	France	Caucasian	40.05	98.46	40.04	98.35	41.44	98.64	41.35	98.64
SRR622457	CEU	Caucasian	65.36	99.82	65.37	99.78	67.19	99.84	67.12	99.84
SRR622458	CEU	Caucasian	58.88	99.36	58.88	99.32	60.94	99.40	60.83	99.40
SRR622459	CEU	Caucasian	58.02	99.45	58.04	99.40	60.06	99.46	59.98	99.46
PAP-MGL0002 -U01-G	Mongolia	Asian	27.81	99.85	27.81	99.82	28.62	99.98	28.57	99.99
HGDP00775	China (Han)	Asian	32.79	98.81	32.78	98.74	33.98	98.94	33.90	98.94
HGDP01308	China (Dai)	Asian	34.26	98.86	34.26	98.78	35.41	99.01	35.34	99.01
PUB-JPN0003 -U01-G	Japan	Asian	60.79	99.97	60.80	99.96	62.96	99.98	62.87	99.99
PUB-JPN0005 -U01-G	Japan	Asian	47.25	99.96	47.25	99.94	48.97	99.98	48.87	99.98
KPGP-00120	Korea	Asian	32.50	99.97	32.51	99.94	33.49	99.99	33.42	99.99
KPGP-00121	Korea	Asian	32.19	99.97	32.19	99.95	32.94	99.99	32.89	99.99
KPGP-00122	Korea	Asian	26.33	99.97	26.33	99.95	27.32	99.99	27.28	99.99
KPGP-00124	Korea	Asian	31.17	99.97	31.17	99.94	31.98	99.99	31.91	99.99
KPGP-00117	Korea	Asian	36.61	99.91	36.62	99.53	37.57	99.94	37.49	99.94

Supplementary Table 30 | All variants compared to GRCh38, GRCh38_C, and KOREFs

a. Variants compared to GRCh38

Nation/tribe	Ethnicity	homozygous SNV	homozygous INDEL	heterozygous SNV	heterozygous INDEL	all variants
Mandenka	African	1,614,344	250,110	3,252,486	423,957	5,540,897
Yoruba	African	1,623,397	259,325	3,287,388	453,110	5,623,220
San	African	1,929,708	299,317	3,330,631	443,792	6,003,448
Mbuti	African	1,834,909	284,177	3,282,740	429,029	5,830,855
Dinka	African	1,640,520	254,011	3,153,108	410,603	5,458,242
Sardinia	Caucasian	1,560,599	253,087	2,507,882	337,472	4,659,040
France	Caucasian	1,512,052	244,518	2,550,429	344,208	4,651,207
CEU	Caucasian	1,495,963	243,410	2,643,275	437,506	4,820,154
CEU	Caucasian	1,517,099	245,221	2,586,786	385,858	4,734,964
CEU	Caucasian	1,483,765	237,393	2,630,607	394,254	4,746,019
Mongolia	Asian	1,602,333	232,843	2,479,567	344,493	4,659,236
China (Han)	Asian	1,650,342	254,437	2,401,103	293,025	4,598,907
China (Dai)	Asian	1,643,907	256,270	2,406,494	300,865	4,607,536
Japan	Asian	1,639,601	267,938	2,516,845	362,831	4,787,215
Japan	Asian	1,668,037	269,589	2,450,423	342,790	4,730,839
Korea	Asian	1,631,396	239,837	2,305,755	292,243	4,469,231
Korea	Asian	1,597,954	230,450	2,367,444	288,357	4,484,205
Korea	Asian	1,601,168	228,671	2,231,534	274,009	4,335,382
Korea	Asian	1,657,144	237,764	2,283,548	276,815	4,455,271
Korea	Asian	1,640,010	248,200	2,335,993	325,122	4,549,325

b. Variants compared to GRCh38_C

Nation/tribe	Ethnicity	homozygous SNV	homozygous INDEL	heterozygous SNV	heterozygous INDEL	all variants
Mandenka	African	1,211,982	243,431	3,305,587	414,358	5,175,358
Yoruba	African	1,231,018	252,663	3,345,092	443,006	5,271,779
San	African	1,516,945	292,609	3,389,637	435,794	5,634,985
Mbuti	African	1,423,658	277,114	3,336,610	420,853	5,458,235
Dinka	African	1,213,904	244,908	3,206,560	401,425	5,066,797
Sardinia	Caucasian	984,396	227,947	2,558,644	327,025	4,098,012
France	Caucasian	914,364	218,931	2,599,928	333,270	4,066,493
CEU	Caucasian	916,802	220,826	2,703,296	422,778	4,263,702
CEU	Caucasian	944,366	222,936	2,643,462	372,847	4,183,611
CEU	Caucasian	907,366	215,316	2,688,540	381,097	4,192,319
Mongolia	Asian	658,202	189,942	2,536,644	329,663	3,714,451
China (Han)	Asian	622,947	201,688	2,449,243	283,102	3,556,980
China (Dai)	Asian	622,883	203,148	2,454,664	290,356	3,571,051
Japan	Asian	624,433	214,155	2,571,845	349,498	3,759,931
Japan	Asian	651,368	215,298	2,503,848	330,550	3,701,064
Korea	Asian	621,435	189,908	2,353,181	280,468	3,444,992
Korea	Asian	581,684	181,304	2,415,107	276,944	3,455,039
Korea	Asian	585,745	178,753	2,280,669	262,940	3,308,107
Korea	Asian	630,821	187,158	2,330,619	265,688	3,414,286
Korea	Asian	625,752	197,653	2,388,942	310,568	3,522,915

c. Variants compared to KOREF_S

Nation/tribe	Ethnicity	homozygous SNV	homozygous INDEL	heterozygous SNV	heterozygous INDEL	all variants
Mandenka	African	1,899,606	271,185	3,301,289	420,873	5,892,953
Yoruba	African	1,919,941	284,415	3,334,640	449,129	5,988,125
San	African	2,188,629	317,078	3,364,703	439,159	6,309,569
Mbuti	African	2,100,096	301,653	3,325,523	425,646	6,152,918
Dinka	African	1,887,255	270,418	3,168,465	406,489	5,732,627
Sardinia	Caucasian	1,728,462	257,330	2,560,749	334,792	4,881,333
France	Caucasian	1,664,474	247,083	2,628,682	343,003	4,883,242
CEU	Caucasian	1,679,236	263,547	2,719,341	431,376	5,093,500
CEU	Caucasian	1,650,211	258,262	2,678,162	384,000	4,970,635
CEU	Caucasian	1,629,051	252,415	2,708,918	389,169	4,979,553
Mongolia	Asian	1,433,902	187,832	2,499,056	331,562	4,452,352
China (Han)	Asian	1,408,738	196,399	2,451,061	288,071	4,344,269
China (Dai)	Asian	1,431,892	203,261	2,458,007	295,599	4,388,759
Japan	Asian	1,399,464	219,953	2,575,275	351,982	4,546,674
Japan	Asian	1,407,595	216,866	2,514,712	334,576	4,473,749
Korea	Asian	1,411,971	188,996	2,377,237	285,905	4,264,109
Korea	Asian	1,383,188	180,090	2,413,482	279,755	4,256,515
Korea	Asian	1,388,544	177,490	2,282,391	265,137	4,113,562
Korea	Asian	1,419,583	184,724	2,350,290	270,957	4,225,554
Korea	Asian	1,415,274	201,750	2,413,606	316,357	4,346,987

d. Variants compared to KOREF_C

Nation/tribe	Ethnicity	homozygous SNV	homozygous INDEL	heterozygous SNV	heterozygous INDEL	all variants
Mandenka	African	1,212,596	206,550	3,292,369	421,829	5,133,344
Yoruba	African	1,237,976	219,861	3,323,619	450,244	5,231,700
San	African	1,505,723	254,670	3,356,014	440,571	5,556,978
Mbuti	African	1,420,095	238,949	3,316,613	427,357	5,403,014
Dinka	African	1,209,682	206,620	3,160,340	407,555	4,984,197
Sardinia	Caucasian	993,587	183,953	2,552,486	335,486	4,065,512
France	Caucasian	922,712	172,431	2,616,202	343,503	4,054,848
CEU	Caucasian	926,900	185,792	2,701,042	431,538	4,245,272
CEU	Caucasian	927,687	182,975	2,649,135	383,506	4,143,303
CEU	Caucasian	903,211	176,639	2,678,229	388,519	4,146,598
Mongolia	Asian	652,322	114,456	2,499,555	328,313	3,594,646
China (Han)	Asian	616,323	115,941	2,441,811	287,953	3,462,028
China (Dai)	Asian	635,841	121,720	2,449,488	295,368	3,502,417
Japan	Asian	576,063	127,970	2,466,876	339,653	3,510,562
Japan	Asian	573,960	123,705	2,450,926	330,414	3,479,005
Korea	Asian	583,492	105,543	2,377,620	284,977	3,351,632
Korea	Asian	554,680	98,501	2,414,149	278,757	3,346,087
Korea	Asian	557,668	96,310	2,283,849	264,161	3,201,988
Korea	Asian	593,228	102,429	2,349,328	269,759	3,314,744
Korea	Asian	590,524	116,896	2,408,198	316,139	3,431,757

Supplementary Table 31 | Variants within the regions shared by GRCh38, GRCh38_C, and KOREFs

a. Variants compared to GRCh38

Nation/tribe	Ethnicity	homozygous SNV	homozygous INDEL	heterozygous SNV	heterozygous INDEL	all variants
Mandenka	African	1,537,873	243,192	2,984,279	410,079	5,175,423
Yoruba	African	1,546,651	252,162	3,008,067	437,903	5,244,783
San	African	1,841,485	291,111	3,045,419	428,629	5,606,644
Mbuti	African	1,753,016	276,634	3,007,386	414,524	5,451,560
Dinka	African	1,567,818	247,302	2,879,582	396,375	5,091,077
Sardinia	Caucasian	1,480,532	245,823	2,247,876	323,930	4,298,161
France	Caucasian	1,437,117	237,741	2,295,762	331,586	4,302,206
CEU	Caucasian	1,413,798	235,749	2,366,980	421,059	4,437,586
CEU	Caucasian	1,435,451	237,311	2,304,493	370,296	4,347,551
CEU	Caucasian	1,406,198	230,185	2,357,944	379,214	4,373,541
Mongolia	Asian	1,523,758	225,799	2,231,015	330,974	4,311,546
China (Han)	Asian	1,575,375	247,925	2,150,845	281,443	4,255,588
China (Dai)	Asian	1,567,327	249,339	2,158,728	289,153	4,264,547
Japan	Asian	1,555,213	259,770	2,233,166	347,895	4,396,044
Japan	Asian	1,585,887	261,995	2,171,749	328,495	4,348,126
Korea	Asian	1,555,627	233,655	2,080,105	281,432	4,150,819
Korea	Asian	1,525,401	224,823	2,137,357	277,678	4,165,259
Korea	Asian	1,532,866	223,272	2,027,996	263,914	4,048,048
Korea	Asian	1,579,741	231,933	2,053,255	266,294	4,131,223
Korea	Asian	1,564,621	241,984	2,102,604	313,656	4,222,865

b. Variants compared to GRCh38_C

Nation/tribe	Ethnicity	homozygous SNV	homozygous INDEL	heterozygous SNV	heterozygous INDEL	all variants
Mandenka	African	1,140,674	234,795	3,029,895	398,762	4,804,126
Yoruba	African	1,158,743	243,701	3,057,738	426,039	4,886,221
San	African	1,435,562	282,503	3,094,711	419,031	5,231,807
Mbuti	African	1,347,609	267,723	3,053,888	404,815	5,074,035
Dinka	African	1,145,701	236,530	2,924,800	385,595	4,692,626
Sardinia	Caucasian	912,707	219,775	2,290,437	311,798	3,734,717
France	Caucasian	846,238	211,278	2,337,491	318,846	3,713,853
CEU	Caucasian	842,235	212,174	2,417,190	404,301	3,875,900
CEU	Caucasian	869,407	214,106	2,352,705	355,499	3,791,717
CEU	Caucasian	836,982	207,081	2,407,010	364,228	3,815,301
Mongolia	Asian	595,351	182,774	2,279,226	314,497	3,371,848
China (Han)	Asian	565,008	195,176	2,191,491	270,050	3,221,725
China (Dai)	Asian	562,927	196,225	2,199,261	277,139	3,235,552
Japan	Asian	559,044	206,148	2,278,808	332,765	3,376,765
Japan	Asian	586,127	207,706	2,215,849	314,543	3,324,225
Korea	Asian	562,965	183,668	2,120,913	268,212	3,135,758
Korea	Asian	526,445	175,743	2,177,998	264,832	3,145,018
Korea	Asian	535,049	173,397	2,070,424	251,501	3,030,371
Korea	Asian	571,978	181,302	2,093,657	253,797	3,100,734
Korea	Asian	566,370	191,396	2,148,469	297,654	3,203,889

c. Variants compared to KOREF_S

Nation/tribe	Ethnicity	homozygous SNV	homozygous INDEL	heterozygous SNV	heterozygous INDEL	all variants
Mandenka	African	1,838,584	261,031	3,193,260	411,299	5,704,174
Yoruba	African	1,855,419	273,126	3,226,650	438,830	5,794,025
San	African	2,121,591	305,602	3,254,073	429,336	6,110,602
Mbuti	African	2,035,747	290,769	3,217,689	416,122	5,960,327
Dinka	African	1,827,074	260,185	3,067,251	397,427	5,551,937
Sardinia	Caucasian	1,663,730	246,752	2,465,532	326,422	4,702,436
France	Caucasian	1,603,905	237,107	2,531,956	334,458	4,707,426
CEU	Caucasian	1,614,403	250,863	2,616,108	420,811	4,902,185
CEU	Caucasian	1,592,455	245,827	2,572,676	373,934	4,784,892
CEU	Caucasian	1,571,221	239,991	2,608,477	379,413	4,799,102
Mongolia	Asian	1,378,650	179,021	2,413,749	323,974	4,295,394
China (Han)	Asian	1,356,821	187,835	2,359,226	280,404	4,184,286
China (Dai)	Asian	1,377,144	194,272	2,367,079	288,021	4,226,516
Japan	Asian	1,344,144	209,132	2,470,649	342,741	4,366,666
Japan	Asian	1,356,151	207,138	2,413,387	325,581	4,302,257
Korea	Asian	1,359,667	181,002	2,292,465	278,932	4,112,066
Korea	Asian	1,331,466	172,387	2,330,028	273,137	4,107,018
Korea	Asian	1,338,598	170,149	2,208,679	259,363	3,976,789
Korea	Asian	1,366,749	176,853	2,265,556	264,238	4,073,396
Korea	Asian	1,361,208	192,930	2,322,623	308,795	4,185,556

d. Variants compared to KOREF_C

Nation/tribe	Ethnicity	homozygous SNV	homozygous INDEL	heterozygous SNV	heterozygous INDEL	all variants
Mandenka	African	1,169,556	199,035	3,177,568	412,169	4,958,328
Yoruba	African	1,192,463	211,369	3,208,543	439,883	5,052,258
San	African	1,457,870	245,947	3,238,385	430,637	5,372,839
Mbuti	African	1,373,409	230,635	3,201,885	417,685	5,223,614
Dinka	African	1,167,547	199,131	3,052,556	398,438	4,817,672
Sardinia	Caucasian	949,611	176,166	2,449,771	327,061	3,902,609
France	Caucasian	883,063	165,329	2,512,690	334,951	3,896,033
CEU	Caucasian	883,635	176,306	2,590,930	420,995	4,071,866
CEU	Caucasian	888,351	173,695	2,537,367	373,554	3,972,967
CEU	Caucasian	864,194	167,370	2,572,881	378,897	3,983,342
Mongolia	Asian	617,152	108,283	2,406,654	320,873	3,452,962
China (Han)	Asian	584,392	110,287	2,342,911	280,331	3,317,921
China (Dai)	Asian	600,804	115,643	2,350,939	287,795	3,355,181
Japan	Asian	542,593	120,860	2,360,342	330,763	3,354,558
Japan	Asian	543,428	117,352	2,347,537	321,787	3,330,104
Korea	Asian	555,357	100,726	2,285,893	278,057	3,220,033
Korea	Asian	527,941	94,040	2,323,498	272,173	3,217,652
Korea	Asian	530,235	92,058	2,203,812	258,373	3,084,478
Korea	Asian	563,836	97,792	2,257,652	262,967	3,182,247
Korea	Asian	560,149	111,283	2,310,227	308,538	3,290,197

Supplementary Table 32 | Differently called variants between KOREF_C and GRCh38

Re-sequenced genome	KOREF_C					GRCh38				
	Total variants	Linkable variants by lift-over	Commonly called variants	Specifically called variants	% of known (dbSNP 144)	Total variants	Lift-overed variants	Commonly called variants	Specifically called variants	% of known (dbSNP 144)
HGDP01286	5,133,344	4,817,523	3,724,661	1,092,862	62.84	5,540,897	5,092,299	3,724,661	1,367,638	90.89
HGDP00936	5,231,700	4,906,223	3,777,871	1,128,352	62.44	5,623,220	5,157,411	3,777,871	1,379,540	90.57
HGDP01036	5,556,978	5,225,218	4,059,097	1,166,121	63.98	6,003,448	5,515,678	4,059,097	1,456,581	89.91
HGDP00982	5,403,014	5,080,484	3,937,532	1,142,952	63.73	5,830,855	5,364,279	3,937,532	1,426,747	90.23
DNK07	4,984,197	4,682,755	3,620,328	1,062,427	63.67	5,458,242	5,023,822	3,620,328	1,403,494	90.59
HGDP01076	4,065,512	3,764,205	2,749,152	1,015,053	60.13	4,659,040	4,216,533	2,749,152	1,467,381	91.85
HGDP00533	4,054,848	3,760,102	2,775,122	984,980	58.46	4,651,207	4,226,021	2,775,122	1,450,899	92.23
SRR622457	4,245,272	3,932,305	2,892,034	1,040,271	56.67	4,820,154	4,353,229	2,892,034	1,461,195	91.78
SRR622458	4,143,303	3,835,419	2,806,641	1,028,778	57.83	4,734,964	4,268,877	2,806,641	1,462,236	91.98
SRR622459	4,146,598	3,850,185	2,857,748	992,437	58.25	4,746,019	4,295,574	2,857,748	1,437,826	92.25
PAP-MGL0002-U01-G	3,594,646	3,321,154	2,581,981	739,173	50.98	4,659,236	4,234,280	2,581,981	1,652,299	92.94
HGDP00775	3,462,028	3,191,805	2,472,583	719,222	48.40	4,598,907	4,182,000	2,472,583	1,709,417	93.33
HGDP01308	3,502,417	3,228,298	2,487,426	740,872	48.95	4,607,536	4,190,235	2,487,426	1,702,809	92.78
PUB-JPN0003-U01-G	3,510,562	3,228,695	2,492,415	736,280	46.55	4,787,215	4,304,660	2,492,415	1,812,245	92.52
PUB-JPN0005-U01-G	3,479,005	3,204,624	2,483,055	721,569	47.18	4,730,839	4,261,777	2,483,055	1,778,722	92.66
KPGP-00120	3,351,632	3,104,933	2,406,100	698,833	49.18	4,469,231	4,094,983	2,406,100	1,688,883	93.17
KPGP-00121	3,346,087	3,106,893	2,446,729	660,164	49.03	4,484,205	4,101,486	2,446,729	1,654,757	93.55
KPGP-00122	3,201,988	2,982,262	2,338,187	644,075	51.35	4,335,382	3,990,411	2,338,187	1,652,224	94.02
KPGP-00124	3,314,744	3,068,830	2,379,991	688,839	49.61	4,455,271	4,068,391	2,379,991	1,688,400	93.37
KPGP-00117	3,431,757	3,169,212	2,439,378	729,834	49.14	4,549,325	4,154,657	2,439,378	1,715,279	92.88

Supplementary Table 33 | Differently called variants excluding repetitive and segmentally-duplicated regions

Re-sequenced genome	KOREF_C				GRCh38			
	Specifically called variants	Variants excluding repetitive and segmentally-duplicated regions	Variants found in dbSNP 144	% of known (dbSNP 144)	Specifically called variants	Variants excluding repetitive and segmentally-duplicated regions	Variants found in dbSNP 144	% of known (dbSNP 144)
HGDP01286	1,092,862	299,091	265,979	88.93	1,367,638	539,509	513,196	95.12
HGDP00936	1,128,352	306,275	270,824	88.43	1,379,540	540,882	513,927	95.02
HGDP01036	1,166,121	329,655	293,693	89.09	1,456,581	574,635	543,375	94.56
HGDP00982	1,142,952	317,551	283,225	89.19	1,426,747	562,818	532,827	94.67
DNK07	1,062,427	293,307	261,979	89.32	1,403,494	549,907	522,865	95.08
HGDP01076	1,015,053	263,759	231,572	87.80	1,467,381	581,224	557,298	95.88
HGDP00533	984,980	244,711	213,247	87.14	1,450,899	580,942	557,868	96.03
SRR622457	1,040,271	254,313	219,226	86.20	1,461,195	577,047	552,595	95.76
SRR622458	1,028,778	250,068	218,577	87.41	1,462,236	574,845	552,937	96.19
SRR622459	992,437	246,130	215,322	87.48	1,437,826	570,444	548,299	96.12
PAP-MGL0002-U01-G	739,173	150,497	125,793	83.59	1,652,299	671,208	646,308	96.29
HGDP00775	719,222	137,325	112,841	82.17	1,709,417	699,766	675,676	96.56
HGDP01308	740,872	144,349	119,116	82.52	1,702,809	692,124	666,145	96.25
PUB-JPN0003-U01-G	736,280	140,793	111,647	79.30	1,812,245	730,151	700,294	95.91
PUB-JPN0005-U01-G	721,569	139,637	111,743	80.02	1,778,722	717,353	689,257	96.08
KPGP-00120	698,833	137,357	113,773	82.83	1,688,883	693,106	667,117	96.25
KPGP-00121	660,164	132,394	109,914	83.02	1,654,757	686,470	661,936	96.43
KPGP-00122	644,075	133,855	112,752	84.23	1,652,224	686,656	662,991	96.55
KPGP-00124	688,839	136,820	114,290	83.53	1,688,400	695,874	670,298	96.32
KPGP-00117	729,834	145,430	118,223	81.29	1,715,279	701,389	674,777	96.21

Supplementary Table 34 | Variant in genic regions compared to GRCh38 and KOREF_C

a. The number of variants found in genic regions compared to GRCh38

Ethnicity / Sample ID		nsSNV		small indels			
				Frame shift		Indels in codon (x3)	
		Homozygous	Heterozygous	Homozygous	Heterozygous	Homozygous	Heterozygous
African	HGDP01286	3,772	8,356	35	75	92	128
	HGDP00936	3,840	8,350	30	96	96	140
	HGDP01036	4,387	8,580	33	94	108	127
	HGDP00982	4,439	8,518	34	81	96	130
	DNK07	3,885	8,059	37	98	77	123
Caucasian	HGDP01076	3,584	6,607	29	66	65	102
	HGDP00533	3,466	6,717	23	73	73	120
	SRR622457	3,498	6,804	38	43	58	106
	SRR622458	3,374	6,567	29	64	48	72
	SRR622459	3,412	6,505	38	51	46	76
Asian	PAP-MGL0002-U01-G	3,651	6,893	25	64	75	122
	HGDP00775	3,769	6,207	33	55	83	108
	HGDP01308	3,683	6,342	28	67	82	94
	PUB-JPN0003-U01-G	3,705	6,710	31	68	85	113
	PUB-JPN0005-U01-G	3,823	6,648	32	75	88	114
	KPGP-00120	3,542	5,755	32	50	56	68
	KPGP-00121	3,525	5,595	28	46	44	61
	KPGP-00122	3,517	5,398	26	41	40	50
	KPGP-00124	3,550	5,616	27	52	54	68
	KPGP-00117	3,679	5,807	26	51	54	71

b. The number of variants found in genic regions compared to KOREF_C

Ethnicity / Sample ID		nsSNV		small indels			
				Frame shift		Indels in codon (x3)	
		Homozygous	Heterozygous	Homozygous	Heterozygous	Homozygous	Heterozygous
African	HGDP01286	2,731	7,999	35	93	71	130
	HGDP00936	2,863	8,039	28	111	85	144
	HGDP01036	3,339	8,141	37	102	88	130
	HGDP00982	3,352	8,071	33	99	83	128
	DNK07	2,751	7,549	26	107	57	132
Caucasian	HGDP01076	2,237	6,203	21	79	49	107
	HGDP00533	2,060	6,402	16	95	45	129
	SRR622457	2,070	6,436	33	58	38	95
	SRR622458	2,032	6,177	23	84	37	80
	SRR622459	2,091	6,129	25	69	37	78
Asian	PAP-MGL0002-U01-G	1,459	6,542	15	80	37	114
	HGDP00775	1,352	5,850	11	74	40	109
	HGDP01308	1,429	5,966	9	83	31	98
	PUB-JPN0003-U01-G	1,363	6,081	14	80	37	112
	PUB-JPN0005-U01-G	1,337	6,362	24	92	28	119
	KPGP-00120	1,188	5,301	10	57	24	68
	KPGP-00121	1,100	5,286	11	56	18	62
	KPGP-00122	1,151	4,983	9	49	14	52
	KPGP-00124	1,285	5,177	8	56	24	67
	KPGP-00117	1,131	5,373	9	51	24	68

Supplementary Table 35 | The number of genes with homozygous variants

Ethnicity / Sample ID		GRCh38			KOREF_C		
		nsSNV	small indel	total	nsSNV	small indel	total
African	HGDP01286	2,669	123	2,742	1,961	100	2,016
	HGDP00936	2,688	117	2,756	2,055	106	2,116
	HGDP01036	3,045	138	3,128	2,319	124	2,393
	HGDP00982	3,012	128	3,083	2,278	115	2,339
	DNK07	2,687	110	2,756	1,946	77	1,998
Caucasian	HGDP01076	2,428	92	2,481	1,503	68	1,546
	HGDP00533	2,374	92	2,435	1,454	54	1,487
	SRR622457	2,388	93	2,440	1,416	64	1,449
	SRR622458	2,376	76	2,418	1,424	58	1,464
	SRR622459	2,335	84	2,382	1,469	59	1,508
Asian	PAP-MGL0002	2,508	100	2,568	1,016	50	1,052
	HGDP00775	2,569	115	2,631	915	48	946
	HGDP01308	2,515	103	2,579	987	38	1,009
	PUB-JPN0003	2,552	112	2,622	933	50	965
	PUB-JPN0005	2,599	115	2,671	913	41	939
	KPGP-00120	2,446	88	2,492	847	33	864
	KPGP-00121	2,440	71	2,477	791	29	808
	KPGP-00122	2,435	66	2,470	837	22	849
	KPGP-00124	2,470	81	2,515	870	32	888
	KPGP-00117	2,521	80	2,563	817	33	838

Supplementary Table 36 | Predicted functionally altered genes by homozygous variants

Ethnicity / Sample ID		GRCh38			KOREF_C		
		nsSNV	small indel	total	nsSNV	small indel	total
African	HGDP01286	368	50	412	336	41	374
	HGDP00936	380	44	413	354	40	384
	HGDP01036	438	48	482	431	47	469
	HGDP00982	442	48	479	426	43	461
	DNK07	416	49	452	359	33	385
Caucasian	HGDP01076	432	45	468	362	29	387
	HGDP00533	404	36	434	327	20	344
	SRR622457	418	50	455	321	36	347
	SRR622458	412	39	442	317	29	342
	SRR622459	397	50	441	362	33	392
Asian	PAP-MGL0002	434	41	473	236	18	254
	HGDP00775	478	47	516	241	15	254
	HGDP01308	454	48	497	244	16	260
	PUB-JPN0003	433	42	469	222	16	236
	PUB-JPN0005	449	47	493	203	21	223
	KPGP-00120	458	50	500	244	18	259
	KPGP-00121	445	39	476	215	16	227
	KPGP-00122	468	40	501	247	12	256
	KPGP-00124	456	45	498	245	17	262
	KPGP-00117	436	42	475	215	18	232

Supplementary Table 37 | Disease term enrichment test for genes predicted to be functionally altered when using GRCh38 but not KOREF_C

Group	Disease term	#Gene	*P*-value	Bonferroni *P*-value
Korean	Adhesion	26	3.20E-08	2.40E-05
	Hypertension	14	3.58E-07	3.00E-04
	Musculoskeletal Diseases	20	3.93E-07	3.00E-04
	Genetic Predisposition to Disease	27	6.77E-07	5.00E-04
	Gestational hypertension	10	6.52E-07	5.00E-04
	Bacterial Infections	12	8.01E-07	6.00E-04
	Myocardial Infarction	14	7.72E-07	6.00E-04
	metabolic syndrome	11	1.86E-06	1.40E-03
	Eclampsia	9	3.09E-06	2.30E-03
	Disease Susceptibility	26	3.15E-06	2.40E-03
	Infarction	13	3.27E-06	2.50E-03
	Bone Diseases	13	4.92E-06	3.70E-03
	Osteonecrosis	5	5.02E-06	3.80E-03
	Coronary Disease	13	6.97E-06	5.20E-03
	Coronary Artery Disease	13	7.27E-06	5.50E-03
	Myocardial Ischemia	13	9.73E-06	7.30E-03
	Collagen Diseases	8	1.35E-05	1.01E-02
	Pre-Eclampsia	8	2.42E-05	1.81E-02
	Dwarfism	7	2.66E-05	1.99E-02
	Gastroschisis	3	2.76E-05	2.07E-02
	Brain Ischemia	8	3.40E-05	2.55E-02
	Mycobacterium Infections	7	3.41E-05	2.56E-02
	Arteriosclerosis	11	3.50E-05	2.62E-02
	Arterial Occlusive Diseases	11	4.32E-05	3.24E-02
	Aggressive Periodontitis	5	4.33E-05	3.25E-02
	Mycobacterial infection	7	4.33E-05	3.25E-02
	Coxa plana	3	4.78E-05	3.58E-02
	Congenital dislocation of hip NOS	4	5.36E-05	4.02E-02
Asian including Korean	Musculoskeletal Diseases	23	1.83E-08	1.28E-05
	Adhesion	26	1.57E-07	1.00E-04
	Bone Diseases	15	4.22E-07	3.00E-04
	Bacterial Infections	12	1.86E-06	1.30E-03
	Myocardial Infarction	14	1.99E-06	1.40E-03
	Collagen Diseases	9	2.83E-06	2.00E-03
	metabolic syndrome	11	4.04E-06	2.80E-03
	Hypertension	13	5.14E-06	3.60E-03
	Genetic Predisposition to Disease	26	9.21E-06	6.40E-03
	Gestational hypertension	9	1.08E-05	7.50E-03
	Disease Susceptibility	25	3.65E-05	2.55E-02
	Infarction	12	3.82E-05	2.67E-02
	Dwarfism	7	4.44E-05	3.10E-02
	Eclampsia	8	4.60E-05	3.21E-02
Caucasian	Musculoskeletal Diseases	17	6.01E-07	3.00E-04
	Common Cold	11	9.83E-06	4.90E-03
	Dystonia Musculorum Deformans	4	1.12E-05	5.60E-03
	Adhesion	17	4.87E-05	2.43E-02
	Respiratory Tract Infections	10	5.19E-05	2.59E-02
	Bone Diseases	10	5.37E-05	2.68E-02
	metabolic syndrome	8	6.48E-05	3.23E-02
	Bacterial Infections	8	1.00E-04	4.99E-02
African	Bone Diseases	11	9.02E-07	4.00E-04
	Musculoskeletal Diseases	13	1.60E-05	6.30E-03
	Collagen Diseases	6	4.63E-05	1.83E-02
	Aggressive Periodontitis	4	8.55E-05	3.38E-02
	metabolic syndrome	7	9.35E-05	3.69E-02
	Adhesion	14	1.00E-04	3.95E-02

Supplementary Table 38 | Pathway enrichment test for genes predicted to be functionally altered when using GRCh38 but not KOREF_C

Group	Pathway	#Gene	*P*-value	Bonferroni *P*-value
Korean	Olfactory transduction	36	4.00E-22	2.16E-20
	ECM-receptor interaction	9	5.16E-07	2.79E-05
	Taste transduction	5	3.00E-04	1.62E-02
	Focal adhesion	9	5.00E-04	2.70E-02
Asian including Korean	Olfactory transduction	36	5.93E-21	3.50E-19
	ECM-receptor interaction	9	1.01E-06	5.96E-05
	Taste transduction	5	4.00E-04	2.36E-02
	Protein digestion and absorption	6	5.00E-04	2.95E-02
	Focal adhesion	9	8.00E-04	4.72E-02
Caucasian	Olfactory transduction	31	7.79E-21	2.57E-19
	ECM-receptor interaction	7	8.52E-06	3.00E-04
	Arrhythmogenic right ventricular cardiomyopathy (ARVC)	5	4.00E-04	1.32E-02
	Hypertrophic cardiomyopathy (HCM)	5	7.00E-04	2.31E-02
	Dilated cardiomyopathy	5	1.10E-03	3.63E-02
African	Olfactory transduction	20	2.50E-12	6.50E-11
	ECM-receptor interaction	6	2.32E-05	6.00E-04

Supplementary Table 39 | Disease associated nsSNVs found against GRCh38 but not KOREF_C

Chr	Pos	Ref	Alt	Gene	A.A. Change	sig	name	acc	Freq. in Korean	Freq. in Asian	Freq. in Caucasian	Freq. in African
1	100206504	T	C	*DBT*	G323S	pathogenic	Intermediate maple syrup urine disease type 2	RCV000012727.21	5/5	4/5	5/5	4/5
1	196690107	C	T	*CFH*	Y402H	pathogenic	Basal laminar drusen	RCV000018016.27	4/5	4/5	4/5	4/5
4	186236880	G	A	*KLKB1*	N124S	pathogenic	Prekallikrein deficiency	RCV000012817.23	2/5	3/5	1/5	4/5
5	35871088	G	A	*IL7R*	I138V	pathogenic	Severe combined immunodeficiency, autosomal recessive, T cell-negative, B cell-positive, NK cell-positive	RCV000015965.24	2/5	0/5	4/5	4/5
5	74685445	T	C	*HEXB*	S62L	pathogenic	Sandhoff disease, infantile type	RCV000004086.1	3/5	5/5	5/5	5/5
7	150999023	T	G	*NOS3*	E298D	pathogenic	Hypertension resistant to conventional therapy	RCV000015056.2	5/5	4/5	0/5	4/5
8	18400806	G	A	*NAT2*	K268R	drug-response	NAT2:N-acetyltransferase 2 (arylamine N-acetyltransferase)	RCV000000760.2	5/5	5/5	3/5	1/5
11	17388025	T	C	*KCNJ11*	E23K	drug response	Exercise stress response, impaired, association with	RCV000009215.1	1/5	3/5	1/5	5/5
12	120999579	A	G	*HNF1A*	G574S	pathogenic	Maturity-onset diabetes of the young, type 3 (MODY3)	RCV000016077.24	5/5	5/5	5/5	4/5
12	121857429	T	C	*HPD*	A33T	pathogenic	4-Alpha-hydroxyphenylpyruvate hydroxylase deficiency	RCV000001643.1	5/5	4/5	3/5	5/5
15	48134287	A	G	*SLC24A5*	A111T	pathogenic	Skin/hair/eye pigmentation, variation in, 4 (SHEP4)	RCV000001552.2	5/5	5/5	0/5	5/5
16	56514589	C	T	*BBS2*	N70S	pathogenic	BARDET-BIEDL SYNDROME 2/6, DIGENIC	RCV000004838.2	5/5	5/5	5/5	5/5
22	18913491	C	T	*PRODH*	Q521R	pathogenic	Proline dehydrogenase deficiency (HYRPRO1)	RCV000004222.4	4/5	5/5	4/5	2/5
